# *Plasmodium* Infection Is Associated with Impaired Hepatic Dimethylarginine Dimethylaminohydrolase Activity and Disruption of Nitric Oxide Synthase Inhibitor/Substrate Homeostasis

**DOI:** 10.1371/journal.ppat.1005119

**Published:** 2015-09-25

**Authors:** Jessica H. Chertow, Matthew S. Alkaitis, Glenn Nardone, Allison K. Ikeda, Aubrey J. Cunnington, Joseph Okebe, Augustine O. Ebonyi, Madi Njie, Simon Correa, Shamanthi Jayasooriya, Climent Casals-Pascual, Oliver Billker, David J. Conway, Michael Walther, Hans Ackerman

**Affiliations:** 1 Laboratory of Malaria and Vector Research, Division of Intramural Research, National Institute of Allergy and Infectious Diseases, National Institutes of Health, Rockville, Maryland, United States of America; 2 Radcliffe Department of Medicine, University of Oxford, John Radcliffe Hospital, Headington Oxford, United Kingdom; 3 Research Technology Branch, Division of Intramural Research, National Institute of Allergy and Infectious Diseases, National Institutes of Health, Rockville, Maryland, United States of America; 4 Section of Paediatrics, Imperial College London, London, United Kingdom; 5 MRC Unit, Fajara, The Gambia; 6 Wellcome Trust Centre for Human Genetics, Oxford, United Kingdom; 7 Wellcome Trust Sanger Institute, Hinxton Cambridge, United Kingdom; 8 London School of Hygiene and Tropical Medicine, Bloomsbury, London, United Kingdom; Albert Einstein College of Medicine, UNITED STATES

## Abstract

Inhibition of nitric oxide (NO) signaling may contribute to pathological activation of the vascular endothelium during severe malaria infection. Dimethylarginine dimethylaminohydrolase (DDAH) regulates endothelial NO synthesis by maintaining homeostasis between asymmetric dimethylarginine (ADMA), an endogenous NO synthase (NOS) inhibitor, and arginine, the NOS substrate. We carried out a community-based case-control study of Gambian children to determine whether ADMA and arginine homeostasis is disrupted during severe or uncomplicated malaria infections. Circulating plasma levels of ADMA and arginine were determined at initial presentation and 28 days later. Plasma ADMA/arginine ratios were elevated in children with acute severe malaria compared to 28-day follow-up values and compared to children with uncomplicated malaria or healthy children (p<0.0001 for each comparison). To test the hypothesis that DDAH1 is inactivated during *Plasmodium* infection, we examined DDAH1 in a mouse model of severe malaria. *Plasmodium berghei* ANKA infection inactivated hepatic DDAH1 via a post-transcriptional mechanism as evidenced by stable mRNA transcript number, decreased DDAH1 protein concentration, decreased enzyme activity, elevated tissue ADMA, elevated ADMA/arginine ratio in plasma, and decreased whole blood nitrite concentration. Loss of hepatic DDAH1 activity and disruption of ADMA/arginine homeostasis may contribute to severe malaria pathogenesis by inhibiting NO synthesis.

## Author Summary

During a malaria infection, the vascular endothelium becomes more adhesive, permeable, and prone to trigger blood clotting. These changes help the parasite adhere to blood vessels, but endanger the host by obstructing blood flow through small vessels. Endothelial nitric oxide (NO) would normally counteract these pathological changes, but NO signalling is diminished malaria. NO synthesis is inhibited by asymmetric dimethylarginine (ADMA), a methylated derivative of arginine that is released during normal protein turnover. We found the ratio of ADMA to arginine to be elevated in Gambian children with severe malaria, a metabolic disturbance known to inhibit NO synthesis. ADMA was associated with markers of endothelial activation and impaired tissue perfusion. In parallel experiments using mice, the enzyme responsible for metabolizing ADMA, dimethylarginine dimethylaminohydrolase (DDAH), was inactivated after infection with a rodent malaria. Based on these studies, we propose that decreased metabolism of ADMA by DDAH might contribute to the elevated ADMA/arginine ratio observed during an acute episode of malaria. Strategies to preserve or increase DDAH activity might improve NO synthesis and help to prevent the vascular manifestations of severe malaria.

## Introduction

Current estimates of world-wide mortality due to malaria range from 367,000 to 755,000 deaths per year, mostly in African children [1,2]. Prompt treatment with parenteral artesunate improves survival in children with severe malaria but mortality remains high in those presenting with complications such as coma or acidosis [3]. Development of effective therapies for these patients will require improved understanding of the pathophysiology of severe malaria.

**Fig 1:**
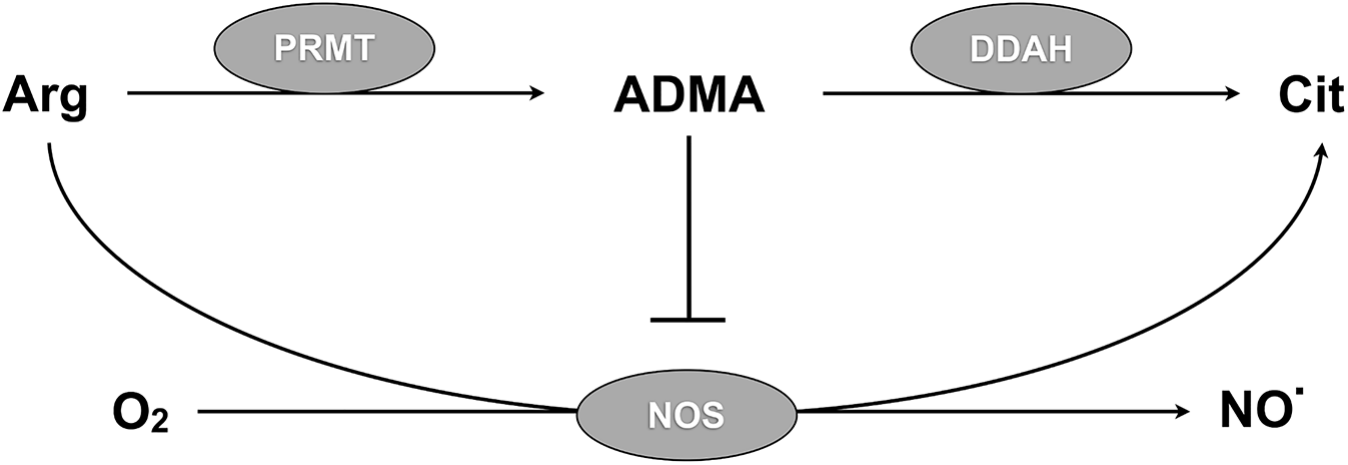
DDAH regulates NO synthesis via ADMA metabolism. Protein arginine methyltransferases (PRMTs) methylate arginine (Arg) residues on proteins to form asymmetric dimethylarginine (ADMA). Proteolysis releases free ADMA that inhibits nitric oxide synthase (NOS). Dimethylarginine dimethylaminohydrolase (DDAH) metabolizes free ADMA to citrulline (Cit) that can be recycled to arginine. Inactivation of DDAH leads to accumulation of ADMA, inhibition of endothelial NO synthesis, and endothelial dysfunction.

Patients with severe malaria exhibit impaired endothelium-dependent vasodilation [4] and reduced nitrite and nitrate concentrations in plasma and urine [5], indicating decreased nitric oxide synthesis. Impaired NO signalling has been implicated in microcirculatory dysfunction [6], loss of blood-brain barrier integrity [7,8] and cytoadherence of infected erythrocytes to the vascular endothelium in mice [9]. Similar pathology has been directly observed in human malaria, but the importance of NO signalling in these processes is less certain [10–13]. NO production by nitric oxide synthase (NOS) is dependent in part on the relative bioavailability of arginine, the NOS substrate, and asymmetric dimethylarginine (ADMA), an endogenous NOS inhibitor released during hydrolysis of proteins that have been methylated by protein arginine methyltransferase [14–16] (Fig 1). By inhibiting NOS, ADMA not only causes vasoconstriction, increased blood pressure, increased systemic vascular resistance and decreased forearm blood flow in vivo [17–19], but also affects adhesion, inflammation, thrombosis, barrier integrity, motility, growth and repair in vitro [20–31]–endothelial functions that are relevant to the pathophysiology of malaria.

Dimethylarginine dimethylaminohydrolase 1 (DDAH1) metabolizes ADMA at a rate inversely proportional to arginine concentration [32] and thus stabilizes the ratio of ADMA to arginine when arginine levels vary [33,34]. In Gambian children, an intronic *DDAH1* polymorphism is associated with susceptibility to severe malaria [35], raising the possibility that DDAH1 might be functionally linked to disrupted ADMA/arginine homeostasis and impaired NO synthesis in severe malaria. In this study, we identify dysregulation of ADMA/arginine homeostasis in Gambian children with severe malaria and hypothesize that ADMA clearance is impaired by hepatic DDAH1 inactivation. To test this hypothesis, we infected mice with *P*. *berghei* ANKA and assessed changes in DDAH1 expression, protein levels and activity in hepatic tissue, a major site of ADMA metabolism [29,36–38].

## Results

### ADMA/Arginine Homeostasis Is Disrupted in Gambian Children with Severe Malaria

We determined ADMA and arginine concentrations in blood plasma obtained from Gambian children with severe or uncomplicated malaria at initial presentation and 28 days later. Healthy afebrile aparasitemic Gambian children served as an additional control group. Baseline characteristics of the study populations are presented in Table 1.

**Table 1:** Baseline clinical characteristics of the study population.

	Healthy Gambian Children	Uncomplicated Malaria	Severe Malaria
Number Enrolled	31	102	96
Age, years	7 [4.5–9.5]	7 [4.0–11.0]	4 [2.7–5.1]^a^ ^,^ ^b^
Weight, kg	18 [13.5–26.5]	19.3 [14.0–26.0]	14.0 [11.0–16.0]^a^ ^,^ ^b^
Temperature, °C	36.8 [36.6–37.0]	38.0 [36.8–38.7]^a^	38.6 [38.1–39.4]^a^ ^,^ ^b^
Respiration rate, breaths/min	-	36 [28–48]	48 [38–60]^b^
Hemoglobin, g/dL	-	11.3 [10.2–12.7]	9.7 [7.0–10.8]^b^
Parasitemia, %	-	2.9 [1.0–6.2]	9.2 [4.8–17.4]^b^
PfHRP2, ng/mL	-	118 [54–226]	249 [133–605]^b^
Glucose, mmol/L	-	6 [4.9–6.7]	6.8 [5.3–8.5]
Lactate, mmol/L	-	2.8 [2.2–4.1]	5.0 [3.2–7.0]^b^
sVCAM, ng/mL	905 [773–1078]	905 [726–1313]	1266 [828–1798]^b^
Haptoglobin, mg/dL	44.5 [15.6–79.9]	1.3 [0.0–49.3]^a^	0.0 [0.0–4.1]^a^

Values are presented as median [interquartile range]. ADMA: asymmetric dimethylarginine, sVCAM: soluble vascular cell adhesion molecule, PfHRP2: *P*. *falciparum* histidine-rich protein 2.

^a^ p < 0.001 compared to healthy Gambian children by Mann-Whitney test.

^b^ p < 0.001 compared to uncomplicated malaria by Mann-Whitney test.

**Fig 2:**
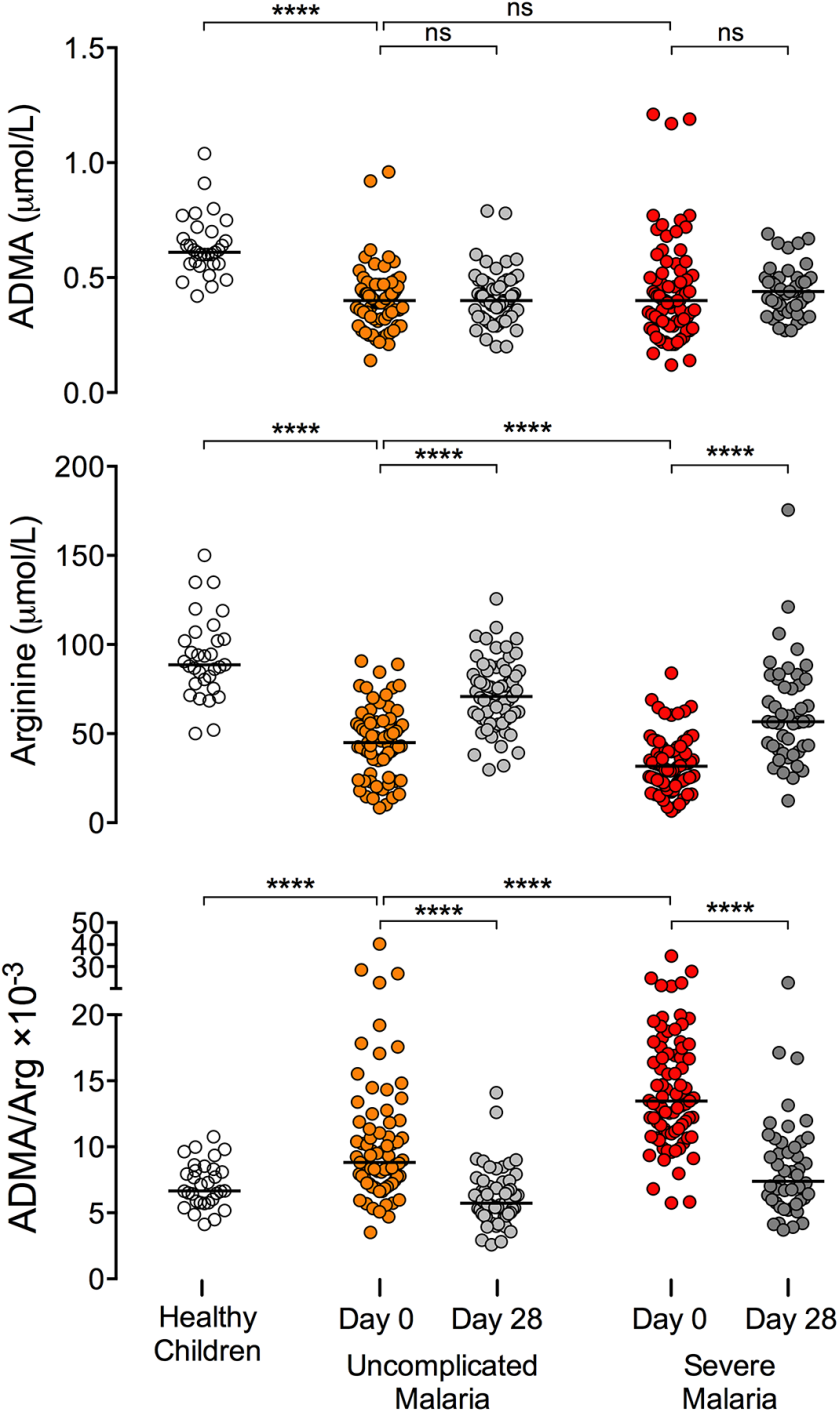
The ADMA/arginine ratio is acutely elevated in African children with severe malaria. ADMA and arginine concentrations were measured in plasma samples collected at the time of presentation (Day 0) and at follow-up visits 28 days later (Day 28) in children with WHO-defined uncomplicated malaria or severe malaria. Healthy Gambian children served as a reference group. Wilcoxon test was used for pair-wise comparison of admission and day 28 mesurements within individuals (47 paired observations from patients with severe malaria; 65 paired observations from patients with uncomplicated malaria). Mann-Whitney test was used to compare patients with severe malaria (n = 81) versus uncomplicated malaria (n = 75) and to compare patients with uncomplicated malaria versus healthy children (n = 31). Each horizontal line depicts the group median. **** p < 0.0001; ns p > 0.05.

Plasma ADMA was lower in children with severe malaria (median [IQR]: 0.40 [0.30–0.51] μmol/L) or uncomplicated malaria (0.40 [0.33–0.47] μmol/L) compared to healthy children (0.61 [0.56–0.69] μmol/L, p < 0.0001 vs. uncomplicated; p < 0.0001 vs. severe; Fig 2 and Table 2). ADMA remained low at the 28-day follow-up visit for patients recovered from malaria. Plasma arginine was profoundly depleted in children with severe malaria compared to children with uncomplicated malaria or healthy children (severe malaria: 31.7 [23.0–40.6] μmol/L; uncomplicated malaria: 45.0 [35.4–55.7] μmol/L, p < 0.0001 vs severe; healthy: 88.7 [79.3–102.5] μmol/L, p < 0.0001 vs severe; Fig 2 and Table 2). By the 28-day follow up visit, plasma arginine concentration increased to 56.7 [42.1–78.9] μmol/L among children who recovered from severe malaria and to 70.8 [58.6–85.1] μmol/L among children who recovered from uncomplicated malaria (p < 0.0001 vs acute, Fig 2), but remained lower than the arginine concentration observed in healthy Gambian children (88.7 μmol/L; p < 0.0001 for either comparison).

**Table 2:** ADMA and arginine concentrations in plasma.

	Healthy Gambian Children	Uncomplicated Malaria		Severe Malaria	
		Day 0	Day 28	Day 0	Day 28
	n = 31	n = 75	n = 65	n = 81	n = 47
ADMA, μmol/L	0.61 [0.56–0.69]	0.40 [0.33–0.47]^a^	0.40 [0.33–0.47]	0.40 [0.30–0.51]^a^	0.44 [0.37–0.50]
Arginine, μmol/L	88.7 [79.3–102.5]	45.0 [35.4–55.7]^a^	70.8 [58.6–85.1]^c^	31.7 [23.0–40.6]^a^ ^,^ ^b^	56.7 [42.1–78.9]^c^
ADMA/Arg x 10^−3^	6.7 [5.8–8.2]	8.8 [7.2–11.6]^a^	5.7 [4.8–6.8]^c^	13.5 [11.2–17.1]^a^ ^,^ ^b^	7.4 [5.9–10.1]^c^

Values are presented as median [interquartile range]. ADMA: asymmetric dimethylarginine. Day 0 was the day of initial presentation to clinic or hospital.

^a^ p < 0.0001 compared to healthy Gambian children by Mann-Whitney test.

^b^ p < 0.0001 compared to uncomplicated malaria by Mann-Whitney test.

^c^ p < 0.0001 compared to admission by Wilcoxon matched-pairs signed-rank test.

ADMA is a competitive inhibitor of NOS, and the ratio of ADMA to arginine determines NOS activity [39]. The ratio of ADMA to arginine was elevated among children with severe malaria (13.5 [11.2–17.1] ×10^−3^) compared to children with uncomplicated malaria (8.8 [7.2–11.6] ×10^−3^, p < 0.0001) or healthy children (6.7 [5.8–8.2] ×10^−3^, p < 0.0001, Fig 2). After recovery from severe malaria, the ADMA/arginine ratio returned to the level observed in healthy Gambian children (recovered from severe malaria: 7.4 [5.9–10.1] ×10^−3^, p < 0.0001 vs. acute; p = 0.25 vs. healthy children; Fig 2 and Table 2). Thus elevation of the ADMA/arginine ratio appears to be an acute metabolic disturbance associated with a symptomatic episode of malaria (modeled in S2 Fig).

### Relationships between Arginine Metabolites and Biomarkers of Malaria Severity

In healthy Gambian children, plasma ADMA concentration was correlated with plasma arginine concentration (r = 0.43, p < 0.05); children with lower arginine tended to have lower ADMA (S1 Fig). In children with uncomplicated malaria, the correlation was stronger (r = 0.59, p < 0.0001) and the slope of the linear regression was steeper (p < 0.0001 vs healthy; S1 Fig). In children with severe malaria, the correlation was stronger (r = 0.77, p < 0.0001) and the slope of the linear regression was steeper still (p < 0.0001 vs uncomplicated; S1 Fig). At the day 28 follow up visit, the relationship between ADMA and arginine (ie, the slopes of the linear regressions) had returned to normal (S1 Fig), though the absolute levels of ADMA and arginine remained lower than in health children.

Lactate is a biomarker of impaired tissue perfusion that is associated with mortality from severe malaria in children [40–43]. In our study, lactate (median [IQR]) was elevated in children with severe malaria (5.0 [3.2–7.0] mmol/L) compared to children with uncomplicated malaria (2.8 [2.2–4.1] mmol/L, p < 0.0001, Table 1). Lactate correlated positively with ADMA among children with severe malaria (r = 0.34, p = 0.004, S3 Fig), implying that tissue perfusion was impaired among those with higher ADMA levels. Lactate did not correlate with arginine (r = 0.16, p = 0.20, S3 Fig) but did correlate with the ADMA/arginine ratio (r = 0.28, p = 0.02, Table 3). In multiple linear regression analysis using ADMA and arginine as explanatory variables, ADMA was positively related to lactate (β = 0.758, p = 0.002) while arginine was negatively and non-significantly related to lactate (β = -0.393, p = 0.09; Table 4).

**Table 3:** Correlation of ADMA with biomarkers of anemia, hemolysis, parasite biomass, endothelial activity, and tissue perfusion among children with severe malaria.

		ADMA		Arg		ADMA/Arg	
	df	r	p	r	p	r	p
Hemoglobin	77	-0.44	0.00004	-0.32	0.004	-0.14	0.21
Haptoglobin	59	-0.29	0.02	-0.24	0.06	-0.05	0.72
HRP2	37	+0.18	0.26	+0.15	0.35	+0.05	0.78
sVCAM	59	+0.60	0.0000002	+0.59	0.0000005	+0.02	0.90
Lactate	69	+0.34	0.004	+0.16	0.20	+0.28	0.02

ADMA, Arg, ADMA/Arg, HRP2 and sVCAM were natural log-transformed. Hemoglobin was normally distributed and was not transformed. Lactate was square root-transformed. Haptoglobin could not be transformed to a normally distributed variable. All correlations were calculated using Pearson’s method, except for correlations with haptoglobin which were calculated using Spearman’s method. A plot of each correlation is presented in the supplement (S3 Fig).

**Table 4:** Multiple linear regression analysis of the relationships between ADMA and arginine and hemoglobin, HRP2, sVCAM, or lactate.

	Explanatory Variables						
	ADMA		Arg		Overall Model		
Dependent Variable	beta	p	beta	p	R^2^	df	p
Hemoglobin	-3.039	0.003	+0.404	0.66	0.20	76	0.0002
HRP2	+0.302	0.53	+0.001	0.99	0.03	36	0.53
sVCAM	+0.413	0.02	+0.332	0.06	0.41	58	0.0000003
Lactate	+0.758	0.002	-0.393	0.09	0.15	68	0.004

ADMA, Arg, HRP2, and sVCAM were natural log-transformed. Lactate was square root-transformed. ADMA and arginine were explanatory variables in four separate linear models predicting hemoglobin, HRP2, sVCAM, or Lactate.

We measured soluble vascular cell adhesion molecule (sVCAM) as a biomarker of endothelial activation. Plasma sVCAM was elevated in children with severe malaria at the time of admission compared to children with uncomplicated malaria or healthy children (severe malaria admission: 1266 [828–1798] ng/mL; acute uncomplicated: 905 [726–1313] ng/mL, p < 0.05 vs severe, healthy children: 905 [773–1078] ng/mL, p < 0.05 vs severe, Table 1). sVCAM returned to normal at day 28 among children who had severe malaria (841 [655–1147] ng/mL, p < 0.001 vs admission). In contrast, the sVCAM level of children with uncomplicated malaria was similar to the level measured in healthy children (p = 0.84). Endothelial activation appears to be a distinctive feature of acute severe malaria.

Soluble VCAM was positively correlated with plasma ADMA in severe malaria patients (r = 0.60, p < 0.0001, Table 3 and S3 Fig). This observation suggests that ADMA, a NOS inhibitor, is associated with endothelial activation and release of sVCAM into circulation. sVCAM was also positively correlated with arginine (r = 0.59, p < 0.0001, Table 3 and S3 Fig). In multiple linear regression analysis, ADMA (β = +0.413, p = 0.02) was more significantly related to sVCAM levels than was arginine (β = +0.332, p = 0.06; Table 4).

Haptoglobin becomes depleted from plasma during acute intravascular hemolysis [44]. Haptoglobin was low at the time of admission in children with severe malaria or uncomplicated malaria (severe: 0 [0–4.1] mg/dL; uncomplicated: 1.3 [0–49.3] mg/dL), and increased by the 28-day follow up visit (severe day 28: 13.8 [1.2–44.3] md/dL, p < 0.0001 vs admission; uncomplicated day 28: 18.7 [0.2–59.4] mg/dL, p < 0.07 vs admission). Admission haptoglobin values, but not day 28 values, were significantly lower than in healthy children (44.5 [15.6–79.9] mg/dL).

Because haptoglobin was undetectable in many children with severe malaria, we analyzed the correlation with ADMA and arginine using Spearman’s method. The correlation between haptoglobin and ADMA was weak (r = -0.29, p = 0.02, Table 3 and S3 Fig), and weaker still with arginine (r = -0.24, p = 0.06, Table 3 and S3 Fig). There were however, moderate negative correlations with hemoglobin, a measure of anemia that may be partially reflective of hemolysis (correlation with Hb and ADMA: r = -0.44, p <0.0001; Hb and Arg r = -0.32, p = 0.004; Table 3 and S3 Fig). Multiple linear regression analysis again revealed that this correlation was primarily due to the association of hemoglobin with ADMA (β = -3.039, p = 0.003) and not with arginine (β = +0.404, p = 0.66; Table 4).

*P*. *falciparum* histidine-rich protein 2 (PfHRP2), a circulating marker of parasite biomass, was higher in children with severe malaria compared to uncomplicated malaria (severe: 249 [133–605] ng/mL vs uncomplicated: 118[54–226] ng/mL, p = 0.0001, Table 1). However, PfHRP2 was not correlated with ADMA or arginine (Table 3; S3 Fig).

### *Plasmodium berghei* Infection Alters Plasma ADMA/Arginine Homeostasis and Blood Nitrite Levels

**Fig 3:**
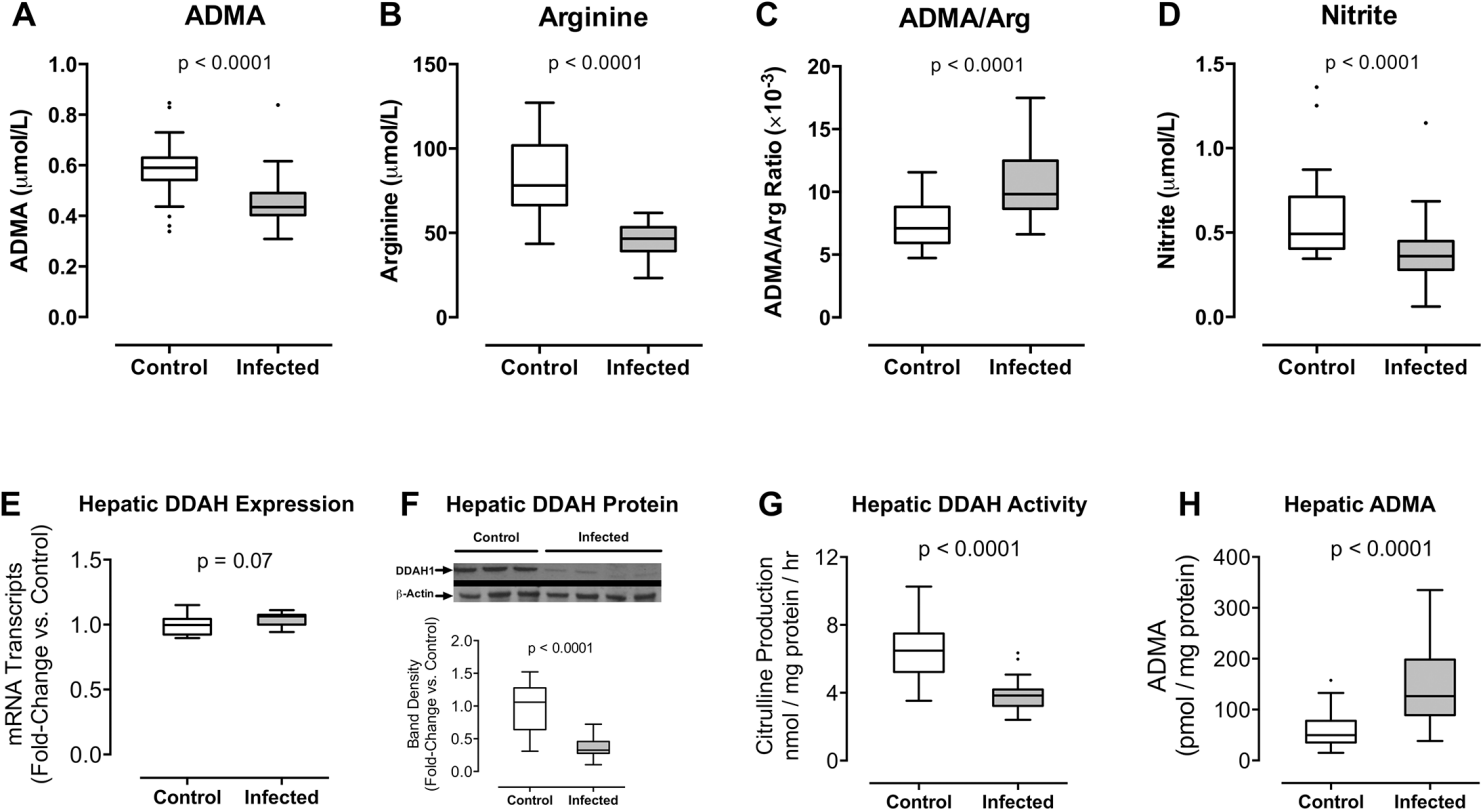
(A-D) *Plasmodium berghei* ANKA infection increases the plasma ratio of ADMA to arginine in mice. HPLC was used to determine **(A)** ADMA, **(B)** arginine concentrations, and **(C)** ADMA/Arg ratio in plasma samples; **(D)** gas phase chemiluminescent assay was used to determine nitrite concentration in blood. Blood was obtained from mice 6 days after inoculation with *P*. *berghei* ANKA (n = 23) and from uninfected control mice (n = 28) in 3 independent experiments. **(E-H)** *Plasmodium berghei* ANKA infection decreases hepatic DDAH activity in mice. **(E)** Quantitative RT-PCR was performed to assess hepatic *Ddah1* expression 6 days after inoculation with *P*. *berghei* ANKA. Values were normalized to *Gapdh* mRNA transcripts and expressed as fold-change vs. control values. Liver samples were obtained from 12 control mice and 12 infected mice representing 2 independent inoculation experiments. **(F)** Western blot was used to detect hepatic DDAH1 protein (38 kDa) in liver tissue obtained from mice 6 days after inoculation with *P*. *berghei* ANKA and from uninfected control mice. β-actin (42 kDa) was used as an internal control. Densitometry was used to quantify DDAH1 band density normalized to β-actin and expressed as fold-change vs. control values. Data are pooled from 12 control mice and 12 infected mice representing 3 independent experiments. **(G)** DDAH activity was assessed by quantification of L-citrulline production by liver homogenates in the presence of saturating concentrations of ADMA substrate (2.5 mM). L-citrulline production was calculated on a per-hour basis and normalized to protein content. **(H)** Intracellular hepatic ADMA was assessed by HPLC in liver homogenates and normalized to protein content. Liver samples were collected from mice 6 days after inoculation with *P*. *berghei* ANKA (n = 25) and from uninfected control mice (n = 28). Results were pooled from 3 independent experiments. Boxes indicate median, 25^th^ and 75^th^ percentiles. Values greater than 1.5 times the IQR are plotted as individual points (Tukey’s method). Mann-Whitney test was used to compare groups.

To determine whether *P*. *berghei* infection was associated with systemic changes in ADMA and arginine, we analyzed plasma from *P*. *berghei* ANKA-infected mice. Similar to Gambian children with malaria, plasma ADMA concentrations were lower in infected animals compared to uninfected controls on day 6 post-inoculation (0.44 [0.40–0.49] vs. 0.59 [0.54–0.63] μmol/L, p < 0.0001, Fig 3A). Arginine concentrations were also lower in infected mice compared to uninfected controls (46.7 [39.2–53.3] vs. 78.1 [66.5–101.9] μmol/L, p<0.0001, Fig 3B). Arginine decreased to a greater extent than ADMA, resulting in an increased ratio of ADMA to arginine among infected mice (9.83 [8.65–12.49] ×10^−3^ vs. 7.10 [5.94–8.81] ×10^−3^, p<0.0001, Fig 3C). The murine findings recapitulated our observations in Gambian children with malaria.

Whole blood nitrite is reflective of NOS activity [45]. We determined nitrite concentrations in whole blood samples from *P*. *berghei* ANKA-infected mice and uninfected controls using a gas-phase chemiluminescent assay. Whole blood nitrite was decreased in infected mice (0.36 [0.28–0.45] μmol/L) compared with uninfected controls (0.49 [0.41–0.71] μmol/L, p = 0.0001, Fig 3D), suggesting that *P berghei* ANKA infection causes a decrease in systemic NO production in mice.

### *Plasmodium berghei* Infection Decreases DDAH1 Abundance in Hepatic Tissue

DDAH1 is highly active in the liver and plays a key role in regulating circulating levels of ADMA [29,33,36–38]. To determine whether severe malaria affects hepatic DDAH1 function, we assessed hepatic *Ddah1* gene expression and protein levels in liver tissue from C57BL/6 mice 6 days after inoculation with *P*. *berghei* ANKA. Using quantitative RT-PCR to assess hepatic *Ddah1* mRNA transcript number relative to *Gapdh*, we found that *Ddah1* gene expression was not changed by *P*. *berghei* ANKA infection (median [IQR] fold change: 1.1 [1.0–1.1], p = 0.07, Fig 3E). In contrast, Western blot analysis revealed a decrease in hepatic DDAH1 protein from *P*. *berghei* ANKA-infected mice compared to uninfected control mice (median [IQR] fold change: 0.33 [0.28–0.46], p < 0.0001, Fig 3F). These data demonstrate that *P*. *berghei* ANKA infection decreases DDAH1 protein abundance by a post-transcriptional mechanism.

### *Plasmodium berghei* Infection Decreases DDAH1 Metabolism of ADMA to Citrulline

To determine the functional impact of hepatic DDAH1 inactivation by *Plasmodium* infection, we quantified ADMA clearance in liver homogenates by measuring the rate of *de novo* citrulline production in the presence of saturating concentrations of ADMA substrate (assay validation presented in S5 Fig). Hepatic ADMA clearance was lower in mice infected with *P*. *berghei* ANKA compared with uninfected controls (infected: 3.83 [3.22–4.19] nmol citrulline × mg protein^-1^ × hr^-1^ vs uninfected control: 6.48 [5.23–7.49] nmol citrulline × mg protein^-1^ × hr^-1^, p < 0.0001, Fig 3G). To assess the impact of *P*. *berghei* infection on hepatic ADMA metabolism, we determined intracellular ADMA concentrations in PBS-perfused liver samples from infected and control mice. ADMA was increased in liver tissue from infected mice compared with uninfected controls (infected: 126.5 [88.9–198.2] pmol/mg protein vs uninfected control: 49.7 [35.3–77.8] pmol/mg protein, p < 0.0001, Fig 3H).

We calculated the correlation between hepatic DDAH activity and hepatic ADMA concentration in healthy mice and found a positive correlation (r = 0.46, p = 0.01), i.e., hepatic DDAH activities were greater in mice that had higher tissue levels of ADMA. This may reflect induction of DDAH activity in response to tissue levels of ADMA. Among *P*. *berghei*-infected mice, the correlation was similar (r = 0.42, p = 0.04) though the tissue levels of ADMA were higher, and the DDAH activities were lower than in uninfected mice (S4 Fig and S1 Table). The partial correlation coefficient between hepatic DDAH activity and hepatic ADMA concentration, accounting for infection status, remained positive (r_part_ = 0.34, p = 0.01). We also calculated the correlation between hepatic DDAH activity and plasma ADMA/Arginine ratio in mice, and found a negative trend, i.e., mice with lower DDAH activity tended to have higher ADMA/Arginine ratios in plasma (r_part_ = -0.23, p = 0.09; S4 Fig and S1 Table).

## Discussion

We have analyzed ADMA and arginine concentrations in plasma from children with severe malaria to determine whether malaria is associated with disruption of ADMA/arginine homeostasis. We found that children with acute severe malaria have uncompensated hypoargininemia, i.e., low arginine with an elevated ADMA/arginine ratio. The hypoargininemia persisted over the 28 days of follow-up, while the ratio of ADMA to arginine returned to normal. We interpret this as a transient inability to metabolize ADMA at a sufficient rate to compensate for low arginine during acute infection. Although plasma ADMA levels were below normal in patients with severe malaria, ADMA was positively correlated with lactate, a biomarker of severity, and sVCAM, a biomarker of endothelial activation, suggesting that higher ADMA levels are associated with adverse pathophysiologic changes. DDAH1 metabolizes ADMA, so we examined changes in DDAH1 activity in mice infected with a *Plasmodium berghei* ANKA, a model of severe malaria. *P berghei* infection caused inactivation of hepatic DDAH1, accumulation of intracellular ADMA in liver tissue, elevation of the ADMA to arginine ratio in plasma, and decreased levels of nitrite in blood. Although these findings in the mouse model cannot be directly extrapolated to human malaria, it raises the possibility that the elevated ADMA/arginine ratio observed in children with severe malaria could be due in part to inactivation of DDAH1.

Our results extend upon a previous report of ADMA and arginine levels in Tanzanian children. Weinberg et al. observed ADMA/arginine ratios of 13.2 [11.1–16.4] ×10^−3^ in cerebral malaria, 12.3 [10.0–15.1] ×10^−3^ in non-cerebral severe malaria, 12.6 [10.7–15.1] ×10^−3^ in moderately severe malaria and 7.1 [5.8–9.0] ×10^−3^ in healthy children [46]. These values are consistent with the ADMA/arginine ratios observed in our study. We found the ADMA/arginine ratio to be significantly greater in children with severe malaria compared to children with uncomplicated malaria, while Weinberg et al found no differences among the ADMA/arginine ratios of cerebral malaria, non-cerebral severe malaria and moderately severe malaria groups. This discrepancy may be explained by the increased severity of the moderately severe malaria group in Weinberg, et al. that differed from our uncomplicated group by the inclusion of patients who could not tolerate oral medication [46]. As a result, these children may have had greater dietary insufficiency of arginine and arginine precursors than our group of uncomplicated malaria patients. Although the plasma arginine concentration was lower in Gambian children with severe malaria compared to the Tanzanian children with cerebral malaria, the rise from admission to day 28 in the Gambian children (31.7 to 56.7 umol/L, an increase of 25 umol/L) was similar to the rise from admission to day 7 in the Tanzanian children (45 umol/l to 70 umol/L, an increase of 25 umol/L).

In both Gambian and Tanzanian children with severe malaria, ADMA and the ADMA/Arg ratio were each correlated with lactate. This could be mediated through the vasoconstrictive or pro-adhesive effects of ADMA on vascular endothelium especially in the setting of hypoarginemia, with subsequent impairment of tissue perfusion leading to anaerobic glycolysis and lactate generation. Inter-individual differences in hepatic blood flow could also be responsible for the strong correlation between ADMA and lactate, since the clearance of each is dependent on hepatic perfusion. Impaired perfusion of liver tissue has been observed in adults with severe malaria [47] and could limit hepatic clearance of plasma ADMA [33,36–38].

In both Gambian and Tanzanian children with severe malaria, ADMA was correlated with biomarkers of endothelial activation (sVCAM and Angiopoietin-2, respectively). This could be through direct effects of ADMA on endothelial cells [48] or via the pro-adhesive effects of ADMA on circulating immune cells that interact with endothelium [49,50].

*P*. *falciparum* histidine-rich protein 2 (PfHRP2) has been previously assessed as a quantitative marker of parasite biomass [51,52]. PfHRP2 did not correlate significantly with ADMA, arginine or the ADMA/arginine ratio among children with severe or uncomplicated malaria (Table 3). Our findings are in agreement with the prior study [46] and together suggest that in children host ADMA metabolism is not determined by parasite biomass.

Arginine depletion and disruption of ADMA/arginine homeostasis have also been observed in adults with moderately severe and severe malaria [53]. In contrast to African children, Indonesian adults with severe malaria demonstrated elevated ADMA [53]. The apparent discrepancy in plasma ADMA in children and adults with severe malaria might be explained by differences in severe malaria pathophysiology observed in older versus younger patients [54]. Changes in plasma ADMA also differ between children and adults with acute sepsis; compared to age-matched healthy controls, ADMA was decreased in pediatric sepsis, but studies of adult sepsis found ADMA to be either unchanged or increased [55–58].

Disruption of ADMA/arginine homeostasis in children with severe malaria could be due to increased protein methylation, accelerated proteolysis of methylated proteins or impaired clearance of free ADMA. One might expect plasma ADMA to be directly elevated during a severe malaria infection, due to the combination of increased release of ADMA from erythrocytes undergoing hemolysis [59] and the impaired activity of hepatic DDAH that we present here. Instead, we observed lower plasma ADMA concentrations in both human and mouse malaria, consistent with prior measurements in children with malaria [46]. This could be due to increased uptake of ADMA from plasma into cellular compartments as has been observed in vitro after LPS, TNF or IL-1 stimulation [60]. In addition, plasma ADMA was strongly correlated with plasma arginine in our study, suggesting that arginine deficiency might lead to lower plasma ADMA. Mechanisms that could potentially link plasma ADMA to plasma arginine are inadequately understood, but might include the requirement for protein-incorporated arginine as the substrate for PRMTs that generate ADMA [16], upregulation of the cationic transporters that allow both ADMA and arginine to cross cell membranes [61,62], and negative feedback of arginine on DDAH activity [32].

DDAH1 is known to regulate ADMA/arginine homeostasis: heterozygous knock-out of DDAH1 in mice increased plasma ADMA, decreased NO-dependent vasodilation, and elevated blood pressure [63]. Conversely, transgenic over-expression of DDAH1 decreased plasma ADMA concentrations, increased urinary nitrites/nitrates and decreased blood pressure [64]. A second DDAH isoform (DDAH2) has been identified [65], but in contrast to *DDAH1*, suppression of *DDAH2* expression did not result in altered plasma ADMA concentrations [34]. The liver expresses *DDAH1* [65] and metabolic tracer studies identified the liver as a major site for clearance of circulating ADMA [37]. Induction of *DDAH1* expression in liver significantly lowered plasma ADMA [33]. Endothelial cell-specific knock-out of DDAH1 revealed hepatic DDAH1 expression not only in hepatic endothelial cells but also in hepatocytes [29,66], which appear to be primarily responsible for systemic ADMA metabolism. Moreover, DDAH1 may regulate the release of ADMA from non-endothelial cell sources that affect local ADMA levels and vascular function. Patients with hepatic failure had elevated plasma levels of ADMA [38,67,68] that decreased after liver transplantation [67]. Conversely, patients with acute rejection of their liver graft had elevated ADMA compared to patients without episodes of rejection [67]. Taken together, results from human patients and animal studies implicate hepatic DDAH1 as a key regulator of circulating ADMA. In severe malaria, renal insufficiency [17], in addition to the hepatic DDAH1 dysfunction we present here, could contribute to dysregulation of ADMA/arginine homeostasis.

*Plasmodium* infection appears to accelerate the degradation of DDAH1 protein in hepatic tissue. Oxidative stress is a potential trigger of DDAH degradation that is present during malaria infection [69,70]. Overexpression of the p22^phox^ subunit of NADPH oxidase in smooth muscle cells increased oxidative stress, decreased DDAH protein levels, decreased DDAH activity and caused accumulation of both intracellular and extracellular ADMA [71]. Treatment of p22^phox^-transfected smooth muscle cells with the proteasome inhibitor epoxomicin raised DDAH protein concentrations and reduced intracellular ADMA, demonstrating that oxidative modification of DDAH protein may target it for degradation by the proteasome. *Plasmodium* infection causes oxidative stress in liver tissue of mice [72], which could be sufficient to accelerate the degradation of DDAH1 in liver endothelium.

The liver may be exposed to reactive oxygen species generated by the increased populations of neutrophils and pigment-laden monocytes found in the hepatic vasculature during malaria infection [72,73]. Increased cell-free heme due to hemolysis promotes neutrophil infiltration and resulting liver damage [72], raising the possibility that hemolysis may contribute to hepatic DDAH dysfunction. Hemolysis may also result in direct release of ADMA into circulation. Human erythrocytes contain total (free plus protein-incorporated) ADMA concentrations in the range of 47.85 ± 1.68 μmol/L, extrapolated from a concentration of 15.95 ± 0.56 μmol/L reported for hydrolysates of erythrocyte samples diluted 1:3 in water [59]. Rat erythrocytes contain a similar concentration of total ADMA, estimated to be 40.6 ± 7.2 μmol/L [74]. Following hemolysis, methylated erythrocyte proteins are exposed to proteases that disproportionately release ADMA relative to arginine [74,75]. Thus hemolysis could both increase ADMA release and inhibit ADMA clearance by promoting DDAH degradation.

Elevation of ADMA relative to arginine favors NOS inhibition because ADMA is a competitive inhibitor of NOS [14]. ADMA also competes with arginine for cellular uptake, which could limit arginine availability for NO synthesis [61]. The intracellular concentration of ADMA in endothelial cells is approximately 10-fold higher than extracellular levels, reaching a concentration of 3–5 μmol/L which is near the K_i_ of eNOS [76,77]. Even small elevations in extracellular ADMA concentration to 2 umol/L had profound effects on brain NOS activity and gene expression profiles of endothelial cells in culture or in mice [78,79]. Impaired NO synthesis has been implicated in impaired vasoregulation, loss of blood-brain barrier integrity and cytoadherence of parasitized erythrocytes to the vascular endothelium during severe malaria. In a mouse model, treatment with an NO-donor improved cerebral microcirculation, reduced cerebral hemorrhages and prevented blood-brain barrier break-down [6,7]. NO synthase inhibition by ADMA downregulates tight junction protein expression [24], which may explain the beneficial effect of NO on endothelial barrier integrity. Impaired NO synthesis is associated with increased adhesion molecule expression [80] and L-NAME (a synthetic NOS inhibitor) increased cytoadherence of parasitized red blood cells to vascular endothelial cells in vitro [9]. Thus, impaired NO signaling may contribute to microhemorrhage, vascular leak and sequestration of parasitized red blood cells observed in children with fatal cerebral malaria [13]. Taken together, these findings suggest that disruption of ADMA/arginine homeostasis could contribute to severe malaria pathogenesis by inhibiting NO synthesis.

Therapeutic strategies that preserve or enhance DDAH activity during *Plasmodium* infection are needed to establish a causal relationship between DDAH degradation and disruption of ADMA/arginine homeostasis. While restoring ADMA/Arginine homeostasis might be necessary to improve endothelial NO synthesis, it might not be sufficient: impaired endothelial NO synthesis is likely to be limited by arginine deficiency and oxidation of tetrahydrobiopterin. NO that is produced will have a limited half-life due to reactions with cell free hemoglobin, superoxide, and other radicals that have increased abundance during malaria infection.

In summary, through clinical observational studies of Gambian children and controlled experiments mice, we have identified hepatic DDAH dysfunction as a potential mechanism disturbing ADMA/Arginine homeostasis and limiting nitric oxide synthesis in severe malaria.

## Methods

### Ethics Statement

Patient enrollment and sample collection were conducted following ethical review and approval by the Gambian Government/MRC Joint Ethics Committee and the Ethics Committee of the London School of Hygiene & Tropical Medicine (SCC 670, SCC 1002, SCC 1003, SCC 1077 & SCC 1113 [healthy children]). Analysis of plasma samples and de-identified clinical data at the National Institutes of Health was exempted from further ethical review by the NIH Office of Subjects Research (Exemption #5161). Written informed consent was provided by a parent or guardian on behalf of children enrolled in the study. Families who declined to participate were provided standard medical care.

Animal studies (described in detail in the supplementary information, S1 Methods) were specifically approved by the National Institutes of Allergy and Infectious Diseases (NIAID) Animal Care and Use Committee (ACUC) under the protocol identification LMVR 18E. The NIAID ACUC complies with the U.S. Government Principles for the Utilization and Care of Vertebrate Animals, the Public Health Service (PHS) Policy on Humane Care and Use of Laboratory Animals, and the Animal Welfare Act.

### Human Subjects and Sample Collection

Children with severe malaria or uncomplicated malaria were enrolled at health centers in a peri-urban area around Fajara, The Gambia as previously described [81]. Enrollment sites included the Royal Victoria Teaching Hospital, the Brikama Health Centre, the MRC Fajara Gate Clinic and the Jammeh Foundation for Peace Hospital in Serekunda.

Acute uncomplicated malaria was defined as asexual *P*. *falciparum* parasitemia of >5000 parasites/μl detected by slide microscopy with an episode of fever (temperature >37.5°C) within the previous 48 hrs and the absence of severe criteria. Acute severe malaria was defined as parasitemia of >5000 parasites/μl, a history of fever and one or more of the following: severe anemia (Hb < 6g/dl), severe acidosis (serum lactate >7 mmol/L), cerebral malaria (Blantyre coma score 2 or less in the absence of hypoglycemia or hypovolemia with the coma lasting for at least 2 hrs), and severe prostration (inability to sit unsupported in children >6 months or inability to suck in children <6 months). Patients with severe malaria were admitted and treated with quinine, and patients with uncomplicated malaria were treated with chloroquine plus sulfadoxine-pyrimethamine according to Gambian Government Treatment Guidelines [82].

Ninety-six children with severe malaria and 102 children with uncomplicated malaria were enrolled during the 2005–2008 malaria seasons. Four milliliters of blood were collected in heparinized vacutainers (BD) at the time of initial presentation and at a follow-up visit 28 days later. Blood samples were immediately refrigerated, placed on ice for transport, and processed within 2 hours of collection. Plasma was frozen at -80**°**C. Three patients with severe malaria died and 65 completed follow-up visits; plasma sample were available from 47 of them. Eighty-five patients with uncomplicated malaria completed follow-up visits; plasma samples were available from 65 of them. Thirty-one healthy, afebrile, aparasitemic Gambian children of similar ages were also studied at a single visit.

### Clinical Laboratory Methods

*P*. *falciparum* parasitemia was determined by bright-field microscopy of giemsa-stained blood smears. 50 fields were counted at high power. Full blood counts were obtained with an automated instrument (Clinical Diagnostics solutions, Inc., Fort Lauderdale, FL, USA). Lactate was measured with a handheld Lactate Pro device (Arkray, Edina, MN, USA). Soluble VCAM (sVCAM) and plasma haptoglobin concentrations were determined by ELISA (sVCAM: R&D Systems, Minneapolis, MN, USA; haptoglobin: Alpco, Salem, NH, USA) according to the manufacturer’s instructions.

*P*. *falciparum* HRP2 was measured in duplicate in plasma by ELISA (Cellabs) according to the manufacturer’s instructions. A standard curve was constructed using serial dilutions of the PfHRP2 standard and run with every plate. Laboratory staff were unaware of clinical status of the subjects.

### Determination of ADMA and Arginine in Human and Mouse Plasma

Each plasma sample was diluted in PBS containing N^G^-monoethyl-L-arginine (MEA) as an internal standard before undergoing solid-phase extraction (Oasis MCX 96-well μElution Plate, Waters Corporation, Milford, MA, USA). The eluted cationic amino acids were dried, resuspended in water, and derivatized with ortho-phthalaldehyde (OPA) in 3-mercaptopropionic acid. Derivatized samples were separated by reverse-phase liquid chromatography over a 1×100 mm C18(2) column (Phenomenex, Torrance, CA, USA) and fluorescence detected at excitation and emission wavelengths of 340nm and 455nm, respectively. Concentrations were determined by integrating peak area with reference to the internal standard (MEA) and daily external standards (Arg, ADMA, and MEA).

### Statistical Analyses

Data are expressed as median and inter-quartile range (IQR). Statistical analyses were performed with GraphPad Prism 6.02 software and the R computing environment. P-values of less than 0.05 were considered significant. Mann-Whitney test was used to compare median values between healthy children and children with uncomplicated or severe malaria. Wilcoxon matched-pairs signed rank test was used to compare acute and recovery values in children with uncomplicated or severe malaria. Correlations were calculated on transformed data using Pearson’s correlation test, except in the case of correlation with haptoglobin in which Spearman’s method was used. When two separate groups were combined, partial correlations were calculated. Multiple linear regression analysis was used to assess the independent contributions of ADMA or arginine to correlations with hemoglobin, HRP2, sVCAM, or lactate. In these linear models, arginine and ADMA were the explanatory variables, and the direction, strength and significance of the association was assessed by the beta value and p-value. Mann-Whitney test was used to compare values between infected mice and uninfected controls. Data are available in supplemental files S1 Data and S2 Data.

## Supporting Information

S1 Table Correlations between Hepatic DDAH Activity and Plasma ADMA/Arg or Hepatic ADMA. Data are presented as Pearson’s correlation coefficients, partial correlation coefficients and p-values. df, degrees of freedom. The results in rows (A) and (B) correspond to S4C and S4D Fig.
(DOCX)

S2 Table Correlations between Hepatic DDAH Densitometry and Plasma ADMA/Arg or Hepatic ADMA. Data are presented as Pearson’s correlation coefficients, partial correlation coefficients and p-values. df, degrees of freedom. The results in rows (C) and (D) correspond to S4C and S4D Fig.
(DOCX)

S1 Fig The relationship between Plasma ADMA and Arginine in Gambian Children. Pearson’s correlation coefficient, *r*, and p-value are provided in the upper left corner of each graph of ADMA and arginine in healthy children (open circles, upper left panel), children with uncomplicated malaria (orange circles, upper middle panel), and children with severe malaria (red circles, upper right panel). Day 28 follow up values are presented in the lower panels. The slopes of the linear regression models are presented in the lower right corner of each graph, along with the p-value for F-tests comparing the slopes of each group as indicated.
(PDF)

S2 Fig Two Alternative Models Depict How the ADMA/Arginine Ratio Changes as Arginine Concentration Falls in Gambian Children. In the No Compensation model, ADMA remains constant as arginine concentration falls, causing the ADMA/Arg ratio to rise according to a reciprocal function. In the Full Compensation model, ADMA is regulated to maintain a constant ADMA/Arg ratio in the setting of falling arginine concentration. The models are based on ADMA and arginine measurements from healthy Gambian children (open circle denotes geometric mean and bars the 95% confidence interval). During acute severe malaria, children exhibit incomplete compensation of ADMA in the setting of hypoargininemia (red circle), but they return to a compensated state by day 28 despite persistent hypoargininemia (dark gray circle). Children with uncomplicated malaria also exhibit incomplete compensation of ADMA in the setting of hypoargininemia and return to a compensated state by day 28 (orange and light gray circles), though the disturbance is less than observed in severe malaria. Arrows indicate the shift from admission to day 28 values.
(PDF)

S3 Fig Correlation Analysis of ADMA or Arginine with Hemoglobin, Haptoglobin, HRP2, sVCAM, or Lactate in Gambian Children with Severe Malaria. X-y plots are presented to support the correlation analyses detailed in Table 3 of the main text. ADMA, Arg, HRP2, sVCAM were natural log-transformed. Lactate was square root-transformed. Hemoglobin and haptoglobin were not transformed. Dotted lines represent the best fits of linear models relating the x and y variables. Refer to Table 4 for multiple linear regression (MLR) analysis of the independent associations of hemoglobin, HRP2, sVCAM and lactate with ADMA or arginine. MLR analysis revealed that these correlations are primarily related to ADMA and not to arginine.
(PDF)

S4 Fig Correlations between Measures of Hepatic DDAH Activity or Abundance and Plasma ADMA/Arginine or Hepatic Tissue Levels of ADMA. Filled circles represent measurements obtained from *P berghei*-infected mice; open circles represent measurements obtained from uninfected control mice. Dotted line represents the linear regression of data from infected mice and dashed line is from uninfected mice. Pearson’s *r* and p-values for the correlations of each group and combined groups are provided in S1 and S2 Tables. The partial correlation provides the combined correlation after accounting for the infected or uninfected status of the animals. A, correlation of plasma ADMA/Arg with hepatic DDAH activity; B, correlation of hepatic ADMA concentration with hepatic DDAH activity; C, correlation of plasma ADMA/Arg with hepatic DDAH western blot densitometry fold change relative to uninfected mice, normalized to GAPDH; and D, correlation of hepatic ADMA with hepatic DDAH western blot densitometry fold change relative to uninfected mice, normalized to GAPDH.
(PDF)

S5 Fig Validation of DDAH Activity Assay. DDAH activity in liver homogenates was assessed by quantifying citrulline production in the presence of saturating concentrations of ADMA substrate (2.5 mmol/L). (A) After addition of 2.5 mmol/L of ADMA to liver homogenate, citrulline increased linearly over time (R^2^ = 0.9972). This confirmed that ADMA metabolism is constant and approximates Vmax kinetics when 2.5 mmol/L ADMA is present. Citrulline production was negligible in the absence of exogenous ADMA (PBS only 0 hr: 0.52 ± 0.19 μM, 2 hr: 0.39 ± 0.07 μM, p > 0.05). (B) Citrulline (25 μmol/L) was stable during a 2 hr incubation at 37°C (0 hr: 26.3 ± 1.6 μM, 2 hr: 25.3 ± 3.0 μM, p > 0.05), demonstrating that citrulline is not degraded or metabolized by enzymes in liver homogenate and is thus a reliable indicator of DDAH activity. (C and D) After addition of 2.5 mmol/L ADMA to liver homogenate, the rate of citrulline production was linear with respect to the protein concentration of liver homogenate in the assay (R^2^ = 0.9995 for line of best fit, 3 replicates for each homogenate dilution).
(PDF)

S1 Methods Supplemental Methods. Detailed description of animal experiments and laboratory methods are provided in the supporting information.
(PDF)

S1 Data De-identified Clinical, HPLC, and ELISA Data from the Children Enrolled in the Study. Data are provided in.csv format for inspection and analysis.
(CSV)

S2 Data Data from *P*. *berghei* ANKA Infections in C57Bl/6 Mice. Data are provided in.csv format for inspection and analysis.
(CSV)
