# Supplementary material for: Plasmodium Infection Is Associated with Impaired Hepatic Dimethylarginine Dimethylaminohydrolase Activity and Disruption of Nitric Oxide Synthase Inhibitor/Substrate Homeostasis
Source: PLoS Pathog. 2015 Sep 25;11(9):e1005119. doi: 10.1371/journal.ppat.1005119 (PMC4583463; doi:10.1371/journal.ppat.1005119)
Supplement: S1 Table — Data are presented as Pearson’s correlation coefficients, partial correlation coefficients and p-values. df, degrees of freedom. The results in rows (A) and (B) correspond to S4C and S4D Fig. (DOCX) [file ppat.1005119.s001.docx]

| **DDAH Activity** | **Infected** | | | **Uninfected** | | | **Combined** | | | **Partial Correlation** | |
| --- | --- | --- | --- | --- | --- | --- | --- | --- | --- | --- | --- |
|  | df | r | p | df | r | p | df | r | p | r_part_ | p |
| **(A) Plasma ADMA/Arg** | 21 | -0.33 | 0.12 | 26 | -0.22 | 0.27 | 49 | -0.54 | 0.00004 | -0.23 | 0.09 |
| **(B) Hepatic ADMA** | 23 | 0.42 | 0.04 | 26 | 0.46 | 0.01 | 51 | -0.23 | 0.10 | 0.34 | 0.01 |
