# Supplementary material for: Plasmodium Infection Is Associated with Impaired Hepatic Dimethylarginine Dimethylaminohydrolase Activity and Disruption of Nitric Oxide Synthase Inhibitor/Substrate Homeostasis
Source: PLoS Pathog. 2015 Sep 25;11(9):e1005119. doi: 10.1371/journal.ppat.1005119 (PMC4583463; doi:10.1371/journal.ppat.1005119)
Supplement: S2 Table — Data are presented as Pearson’s correlation coefficients, partial correlation coefficients and p-values. df, degrees of freedom. The results in rows (C) and (D) correspond to S4C and S4D Fig. (DOCX) [file ppat.1005119.s002.docx]

| **DDAH Densitometry** | **Infected** | | | **Uninfected** | | | **Combined** | | | **Partial Correlation** | |
| --- | --- | --- | --- | --- | --- | --- | --- | --- | --- | --- | --- |
|  | df | r | p | df | r | p | df | r | p | r_part_ | p |
| **(C) Plasma ADMA/Arg** | 7 | 0.03 | 0.94 | 10 | 0.54 | 0.07 | 19 | -0.18 | 0.44 | 0.30 | 0.18 |
| **(D) Hepatic ADMA** | 9 | -0.32 | 0.33 | 10 | -0.10 | 0.76 | 21 | -0.47 | 0.02 | -0.19 | 0.42 |
