# Supplementary figures and images for: Plasmodium Infection Is Associated with Impaired Hepatic Dimethylarginine Dimethylaminohydrolase Activity and Disruption of Nitric Oxide Synthase Inhibitor/Substrate Homeostasis
Source: PLoS Pathog. 2015 Sep 25;11(9):e1005119. doi: 10.1371/journal.ppat.1005119 (PMC4583463; doi:10.1371/journal.ppat.1005119)

### Healthy

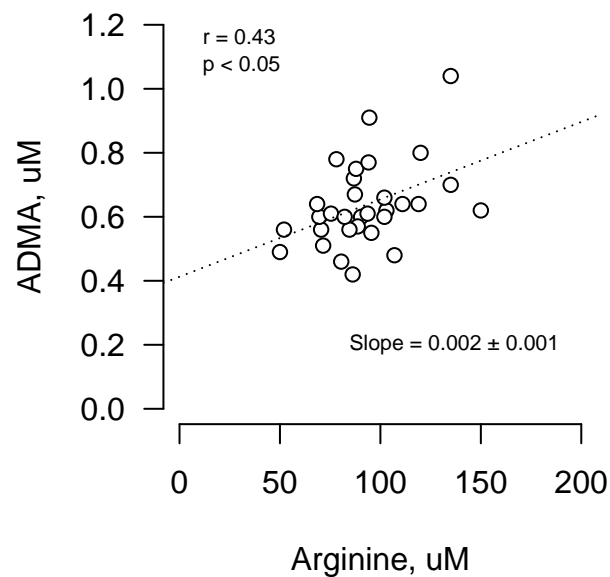

### Uncomplicated Malaria Admission

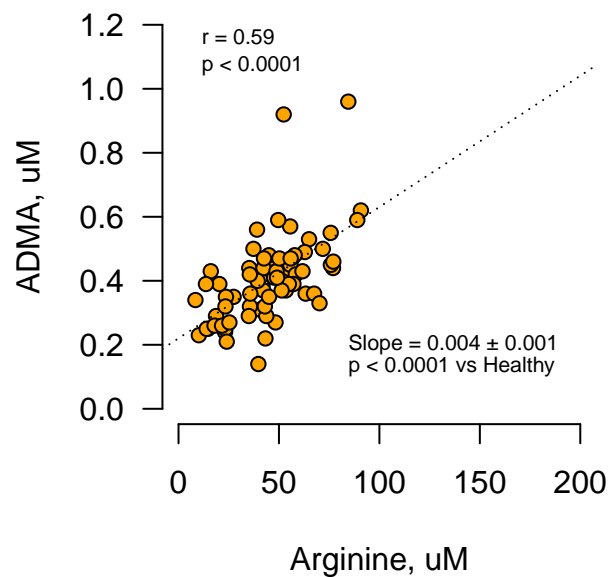

### Severe Malaria Admission

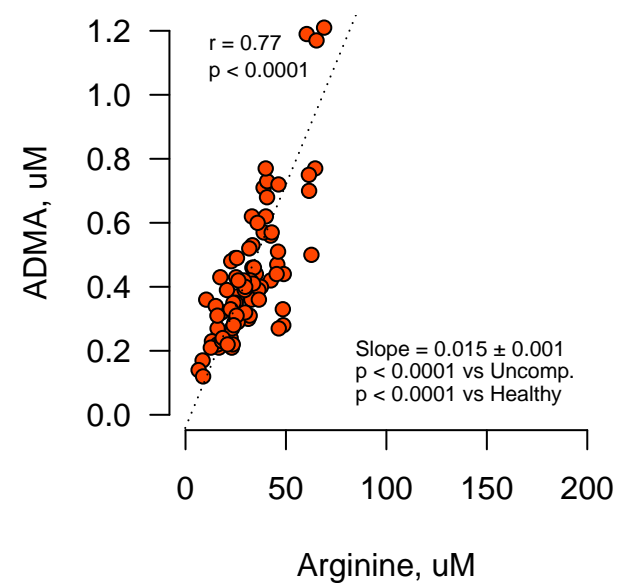

### Day 28

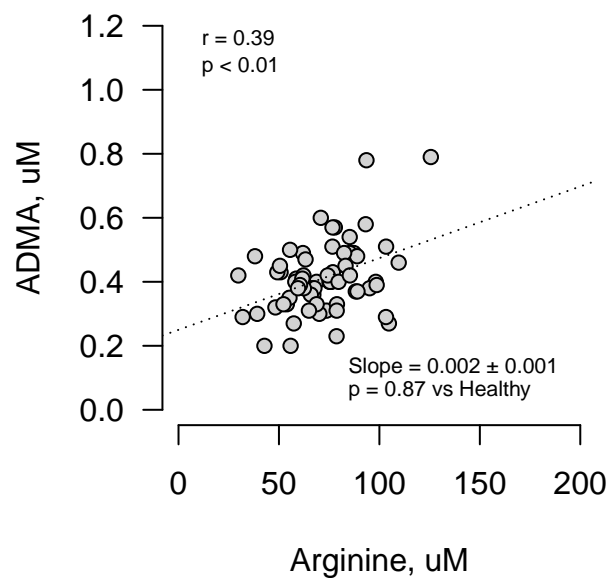

### Day 28

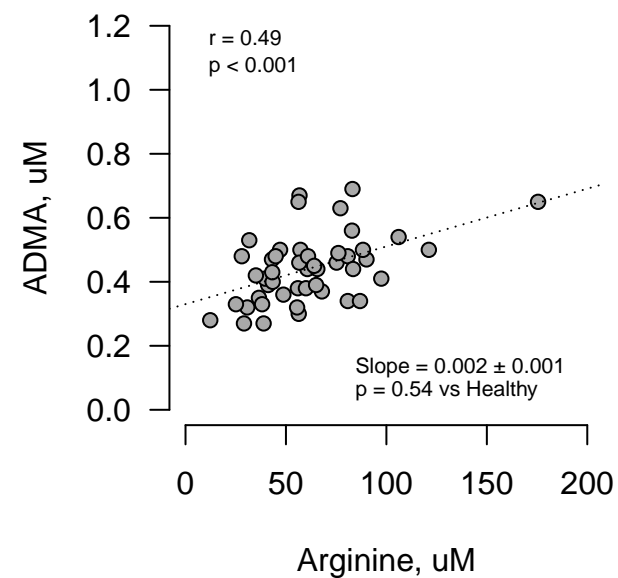

Supplement: S1 Fig — Pearson’s correlation coefficient, r, and p-value are provided in the upper left corner of each graph of ADMA and arginine in healthy children (open circles, upper left panel), children with uncomplicated malaria (orange circles, upper middle panel), and children with severe malaria (red circles, upper right panel). Day 28 follow up values are presented in the lower panels. The slopes of the linear regression models are presented in the lower right corner of each graph, along with the p-value for F-tests comparing the slopes of each group as indicated. (PDF) [file ppat.1005119.s003.pdf]

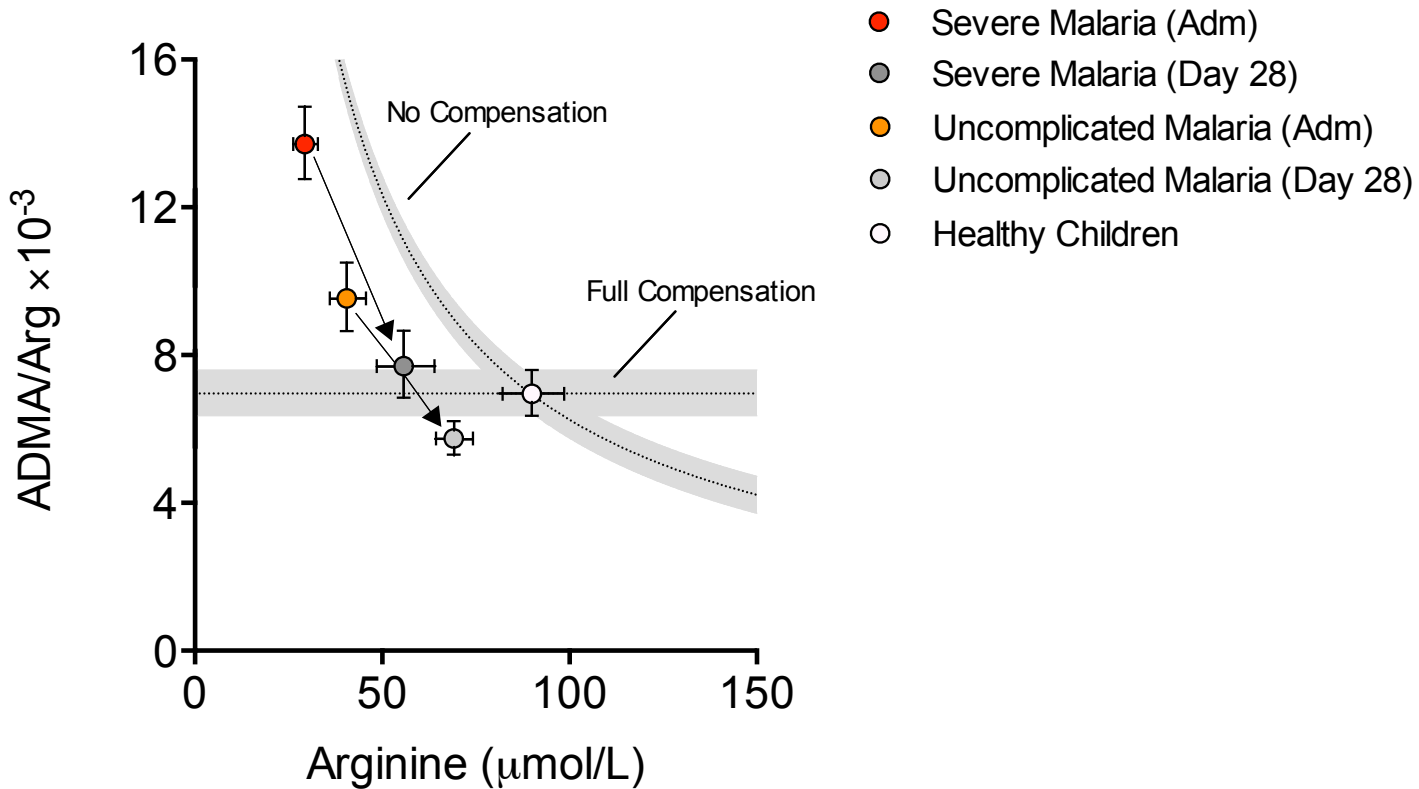

Supplement: S2 Fig — In the No Compensation model, ADMA remains constant as arginine concentration falls, causing the ADMA/Arg ratio to rise according to a reciprocal function. In the Full Compensation model, ADMA is regulated to maintain a constant ADMA/Arg ratio in the setting of falling arginine concentration. The models are based on ADMA and arginine measurements from healthy Gambian children (open circle denotes geometric mean and bars the 95% confidence interval). During acute severe malaria, children exhibit incomplete compensation of ADMA in the setting of hypoargininemia (red circle), but they return to a compensated state by day 28 despite persistent hypoargininemia (dark gray circle). Children with uncomplicated malaria also exhibit incomplete compensation of ADMA in the setting of hypoargininemia and return to a compensated state by day 28 (orange and light gray circles), though the disturbance is less than observed in severe malaria. Arrows indicate the shift from admission to day 28 values. (PDF) [file ppat.1005119.s004.pdf]

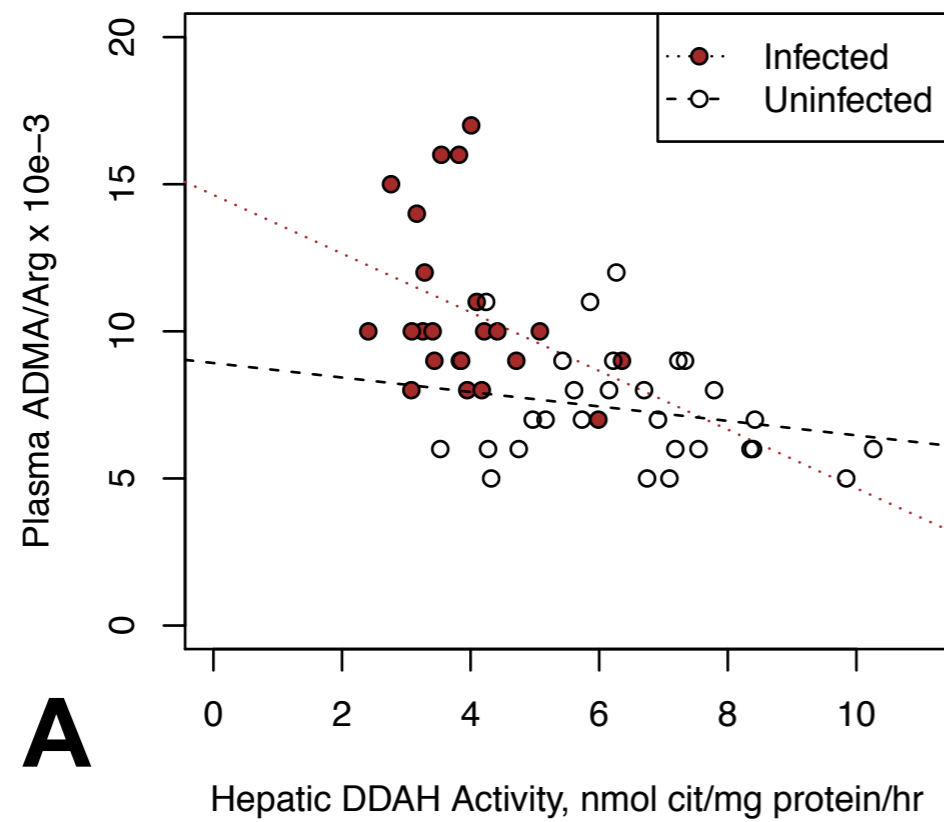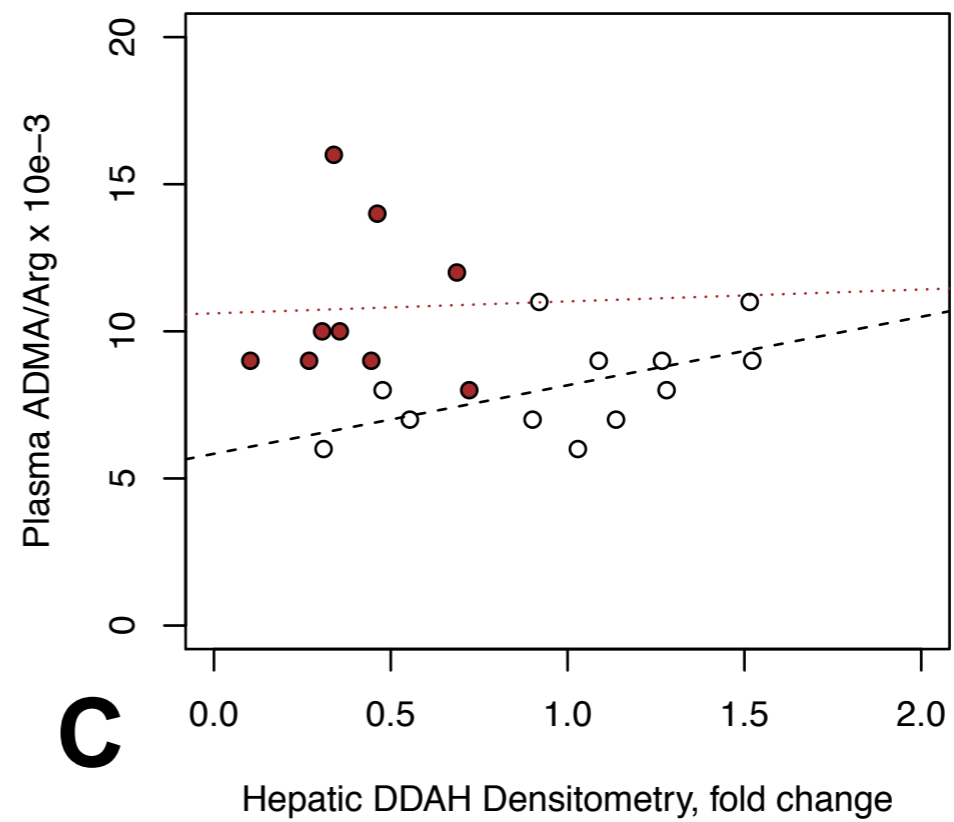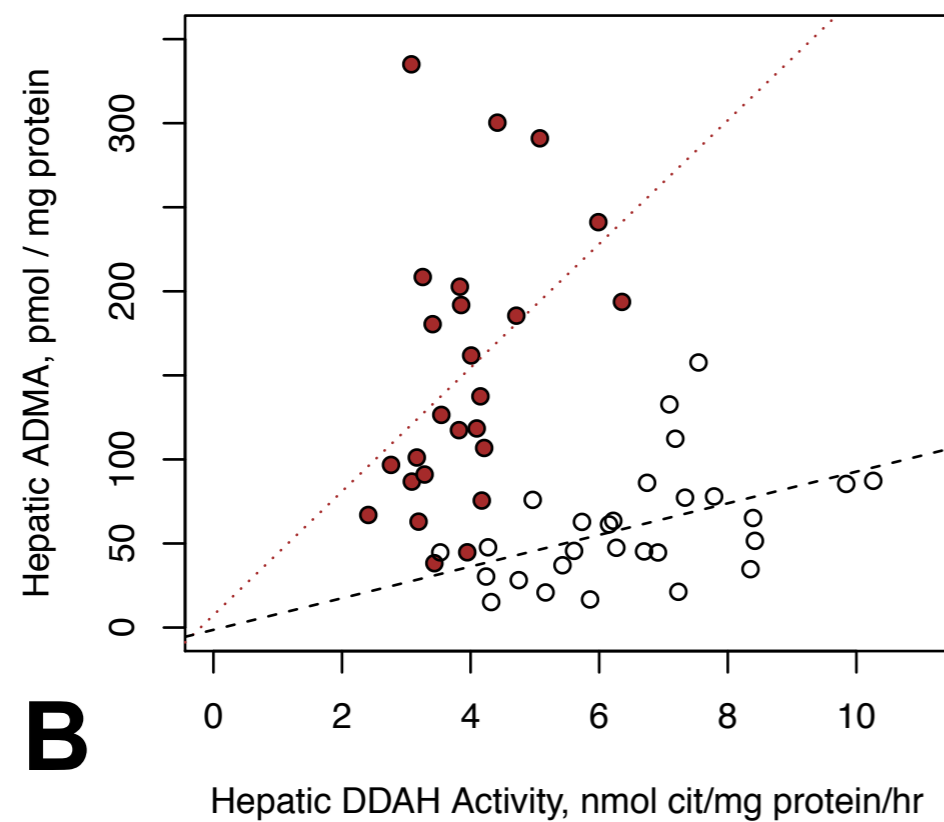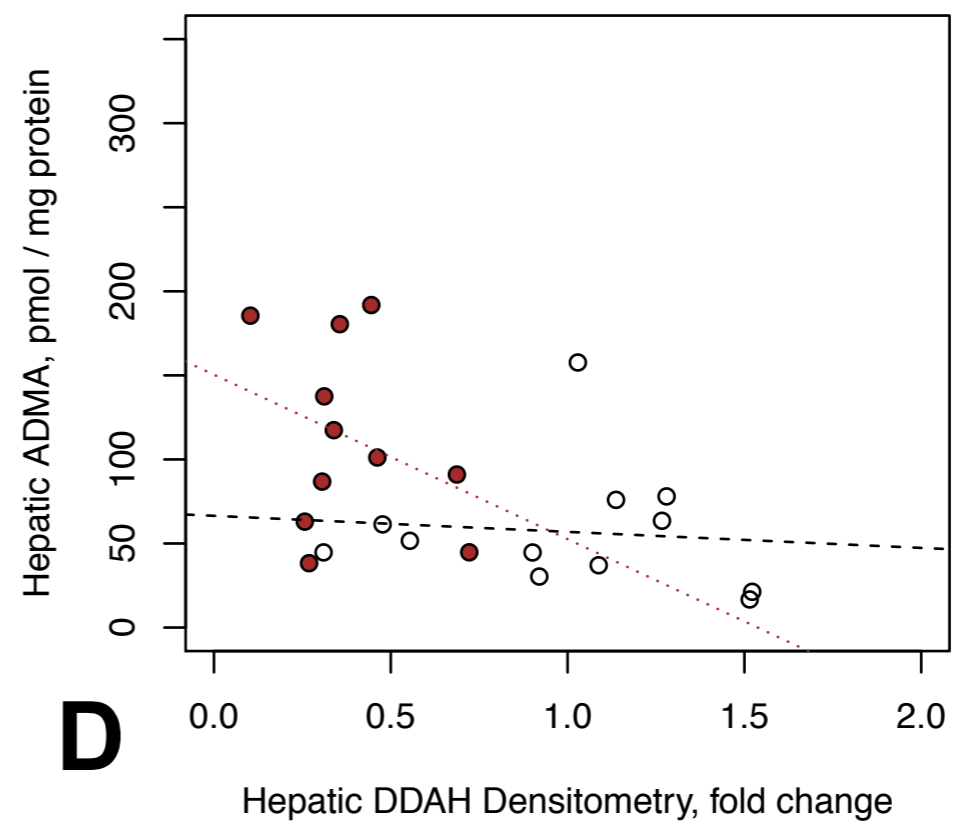

Supplement: S4 Fig — Filled circles represent measurements obtained from P berghei-infected mice; open circles represent measurements obtained from uninfected control mice. Dotted line represents the linear regression of data from infected mice and dashed line is from uninfected mice. Pearson’s r and p-values for the correlations of each group and combined groups are provided in S1 and S2 Tables. The partial correlation provides the combined correlation after accounting for the infected or uninfected status of the animals. A, correlation of plasma ADMA/Arg with hepatic DDAH activity; B, correlation of hepatic ADMA concentration with hepatic DDAH activity; C, correlation of plasma ADMA/Arg with hepatic DDAH western blot densitometry fold change relative to uninfected mice, normalized to GAPDH; and D, correlation of hepatic ADMA with hepatic DDAH western blot densitometry fold change relative to uninfected mice, normalized to GAPDH. (PDF) [file ppat.1005119.s006.pdf]

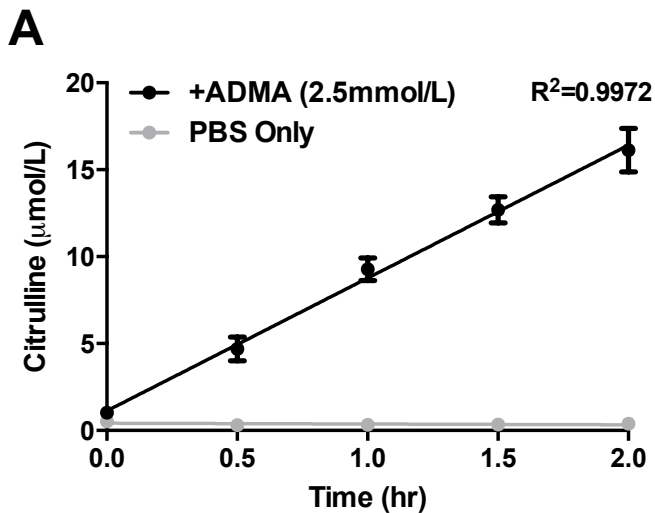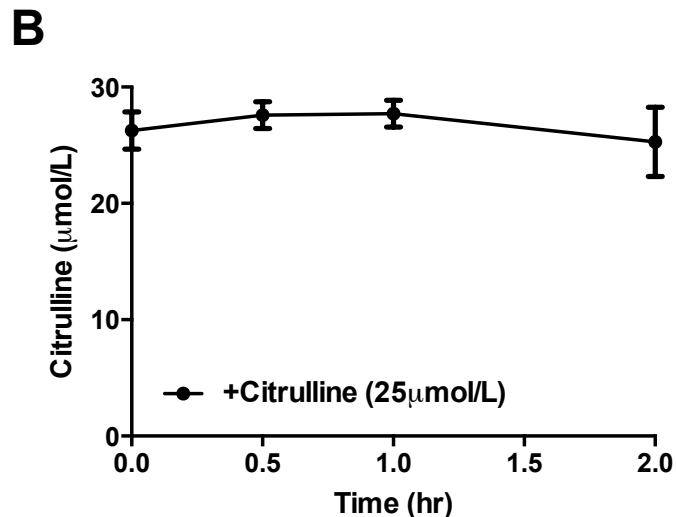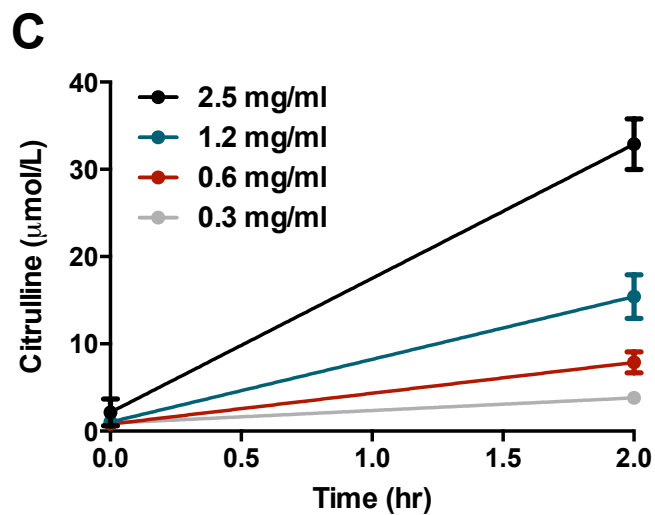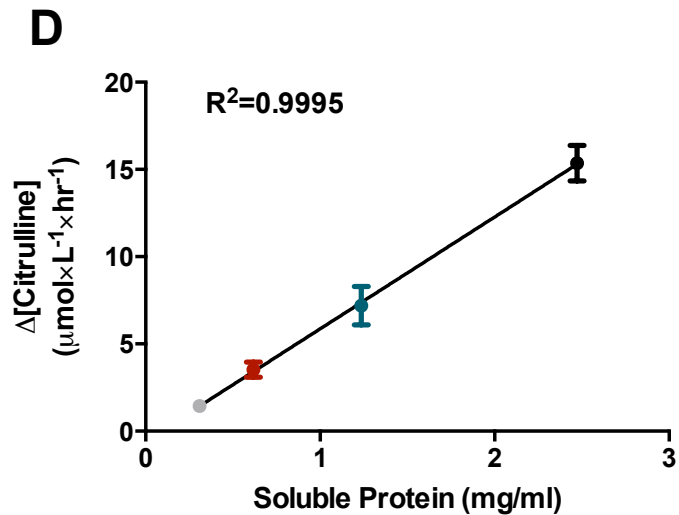

Supplement: S5 Fig — DDAH activity in liver homogenates was assessed by quantifying citrulline production in the presence of saturating concentrations of ADMA substrate (2.5 mmol/L). (A) After addition of 2.5 mmol/L of ADMA to liver homogenate, citrulline increased linearly over time (R2 = 0.9972). This confirmed that ADMA metabolism is constant and approximates Vmax kinetics when 2.5 mmol/L ADMA is present. Citrulline production was negligible in the absence of exogenous ADMA (PBS only 0 hr: 0.52 ± 0.19 μM, 2 hr: 0.39 ± 0.07 μM, p > 0.05). (B) Citrulline (25 μmol/L) was stable during a 2 hr incubation at 37°C (0 hr: 26.3 ± 1.6 μM, 2 hr: 25.3 ± 3.0 μM, p > 0.05), demonstrating that citrulline is not degraded or metabolized by enzymes in liver homogenate and is thus a reliable indicator of DDAH activity. (C and D) After addition of 2.5 mmol/L ADMA to liver homogenate, the rate of citrulline production was linear with respect to the protein concentration of liver homogenate in the assay (R2 = 0.9995 for line of best fit, 3 replicates for each homogenate dilution). (PDF) [file ppat.1005119.s007.pdf]
