# Supplementary material for: Plasmodium Infection Is Associated with Impaired Hepatic Dimethylarginine Dimethylaminohydrolase Activity and Disruption of Nitric Oxide Synthase Inhibitor/Substrate Homeostasis
Source: PLoS Pathog. 2015 Sep 25;11(9):e1005119. doi: 10.1371/journal.ppat.1005119 (PMC4583463; doi:10.1371/journal.ppat.1005119)
Supplement: S3 Fig — X-y plots are presented to support the correlation analyses detailed in Table 3 of the main text. ADMA, Arg, HRP2, sVCAM were natural log-transformed. Lactate was square root-transformed. Hemoglobin and haptoglobin were not transformed. Dotted lines represent the best fits of linear models relating the x and y variables. Refer to Table 4 for multiple linear regression (MLR) analysis of the independent associations of hemoglobin, HRP2, sVCAM and lactate with ADMA or arginine. MLR analysis revealed that these correlations are primarily related to ADMA and not to arginine. (PDF) [file ppat.1005119.s005.pdf]

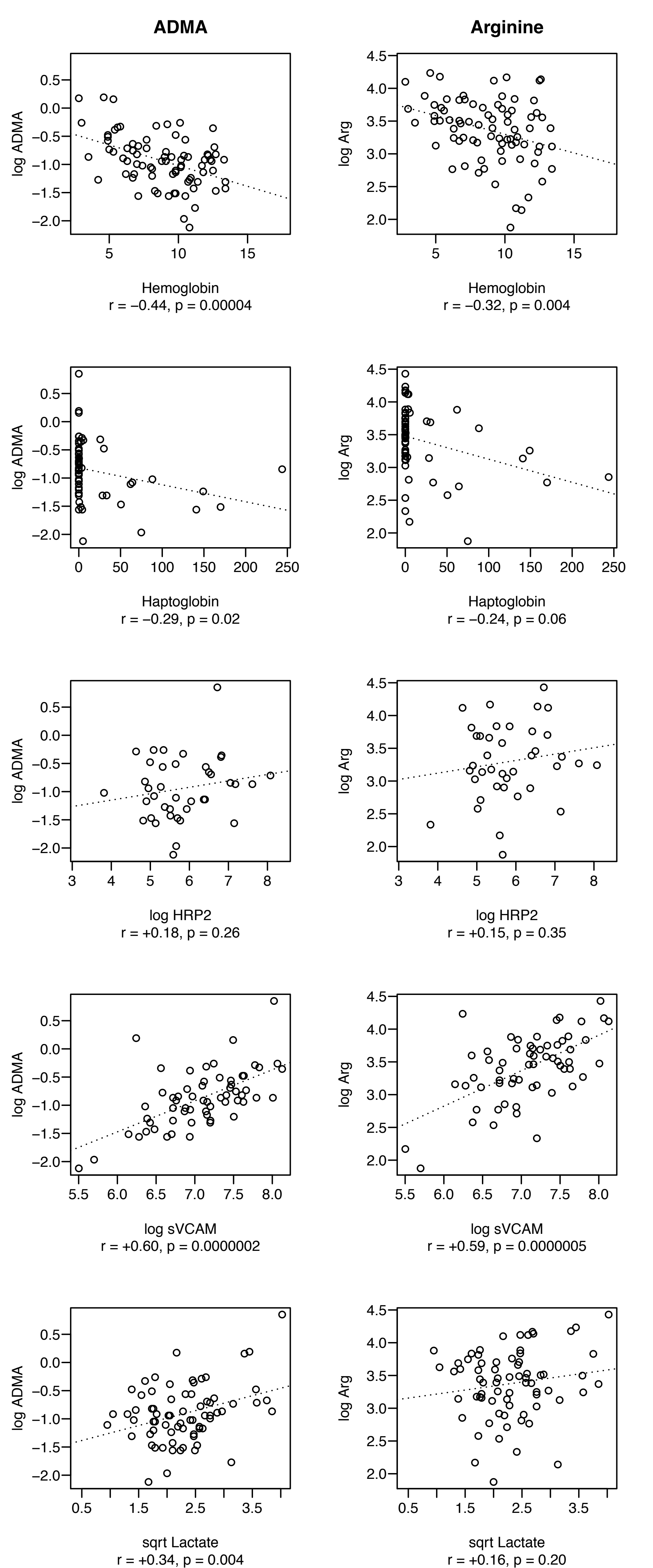

ADMA
Arginine
log ADMA
log Arg
Hemoglobin
r = −0.44, p = 0.00004
Hemoglobin
r = −0.32, p = 0.004
Haptoglobin
r = −0.29, p = 0.02
Haptoglobin
r = −0.24, p = 0.06
log HRP2
r = +0.18, p = 0.26
log HRP2
r = +0.15, p = 0.35
log sVCAM
r = +0.60, p = 0.0000002
log sVCAM
r = +0.59, p = 0.0000005
sqrt Lactate
r = +0.34, p = 0.004
sqrt Lactate
r = +0.16, p = 0.20
