# Supplementary material for: Plasmodium Infection Is Associated with Impaired Hepatic Dimethylarginine Dimethylaminohydrolase Activity and Disruption of Nitric Oxide Synthase Inhibitor/Substrate Homeostasis
Source: PLoS Pathog. 2015 Sep 25;11(9):e1005119. doi: 10.1371/journal.ppat.1005119 (PMC4583463; doi:10.1371/journal.ppat.1005119)
Supplement: S1 Methods — Detailed description of animal experiments and laboratory methods are provided in the supporting information. (PDF) [file ppat.1005119.s008.pdf]

SUPPORTING INFORMATION

Plasmodium Infection Is Associated with Impaired Hepatic Dimethylarginine Dimethylaminohydrolase Activity and Disruption of Nitric Oxide Synthase Inhibitor/Substrate Homeostasis by Chertow, Alkaitis, et al. (2015)

SUPPLEMENTAL METHODS

Determination of ADMA and Arginine in Human Plasma

Plasma was thawed and 10µl was diluted in 35µl PBS (Lonza, Walkersville, MD, USA) containing N^G-monoethyl-L-arginine (MEA) (Enzo Life Sciences, Farmingdale, NY, USA) as an internal standard. Oasis MCX 96-well µElution Plates (Waters Corporation, Milford, MA, USA) were conditioned with elution buffer prepared from ultrapure water (Milli-Q Synthesis system, Millipore Corporation, Billerica, MA, USA), HPLC-grade methanol (Fisher Scientific, Pittsburgh, PA, USA) and 28% ammonium hydroxide (Sigma-Aldrich, St. Louis, MO, USA) in a ratio of 5:4:1 H_2O:MeOH:NH_4OH and equilibrated with methanol. Samples were loaded, washed with 0.1 N HCl (Sigma-Aldrich, St. Louis, MO, USA) followed by methanol, eluted with elution buffer, dried by vacuum centrifugation at 45°C and reconstituted in 10µl ultrapure water. Reconstituted samples were mixed 1:1 with a derivatization reagent containing 7.46 mM ortho-phthalaldehyde (Sigma-Aldrich, St. Louis, MO, USA) and 27.5 mM 3-mercaptopropionic acid (Sigma-Aldrich, St. Louis, MO, USA) in 200 mM potassium tetraborate (Sigma-Aldrich, St. Louis, MO, USA), pH 9.4, and 3 µl was injected onto a 2×100 mm C18(2) column with 100 Å pores and 5 µm particles (Phenomenex, Torrance, CA, USA). Chromatography was performed isocratically at a flow-rate of 0.3 ml/min. The mobile phase consisted of 25 mM ammonium phosphate (Sigma-Aldrich, St. Louis, MO, USA) and 9% acetonitrile (Fisher Scientific, Pittsburgh, PA, USA) in ultrapure water from a Milli-Q Synthesis system (EMD Millipore, Billerica, MA, USA). Derivatized analytes were detected by on-line fluorescence detection using excitation and emission wavelengths of 340 nm and 455nm respectively. Following elution of arginine, sensitivity was increased 32-fold for the detection of ADMA by increasing the gain on the fluorescence detector. Following elution of ADMA, sensitivity was returned to normal and a second solvent containing 50 % acetonitrile and 50 % ultrapure water was introduced at 12 % to accelerate elution of the monoethylarginine internal standard. The column was then washed with 100 % solvent B for 5 minutes and re-equilibrated prior to injection of the following sample. Chemstation revision B was used for data acquisition and analysis. Analyte peaks were integrated and quantified by comparison to the internal standard. Inter- and inter-day variation was < 5% for repeat analysis of human samples using this assay.

Animals, Housing and Diet

Animal experiments were performed at the National Institutes of Health (NIAID Comparative Medicine Branch) using protocols approved by the NIH ACUC under the identification ASP LMVR 18E. 10-week-old C57BL/6J male mice were obtained from the Jackson Laboratory (Bar Harbor, ME, USA) and 10-week-old Swiss Webster carriers for parasite expansion were obtained from Charles River Laboratories (Frederick, MD, USA). Mice were housed in temperature-controlled cages maintained at 20-22ºC with a 12hr/12hr light-dark cycle and free access to water and autoclaved rodent feed pellets (Teklad Global 18% Protein Extruded Rodent Diet, 2018SX, Harlan Laboratories, Frederick, MD, USA). Mouse model experiments were replicated at the Wellcome Trust Sanger Institute under personal (PIL 80/12137) and project licenses (PPL 80/2158) issued by the UK Home Office in accordance with the Animals (Scientific Procedures) Act of 1986. Protocols reviewed by the Wellcome Trust Sanger Institute ethics committee. For this experiment, 10-week-old male C57BL/6J experimental mice and Tuck Ordinary outbred mice carriers for parasite expansion were obtained from Harlan Laboratories (London, UK).

Mice were housed in temperature-controlled cages maintained at 20-22ºC with a 12hr/12hr light-dark cycle and free access to water and autoclaved rodent feed pellets (Teklad Global 18% Protein Extruded Rodent Diet, 2018SX, Harlan Laboratories). Experimental groups were assigned in an

alternating manner such that every experimental group was represented in each cage of communally-housed littermates.

Parasites, Inoculations and Parasitemia Assessment

P. berghei ANKA parasites, clone RMgm-29 [63], were expanded from frozen stocks by intraperitoneal injection of carrier mice. After four days of parasite expansion, whole blood was obtained from the abdominal inferior vena cava under terminal anesthesia. Cohorts of experimental mice were infected by intraperitoneal injection of 1×10^6 infected erythrocytes diluted in 200 µl of PBS (Lonza, Walkersville, MD, USA). This protocol results in blood-stage infection, bypassing the liver stage of the parasite's life-cycle. Blood-stage parasitemia was monitored by examination of Giemsa-stained blood smears obtained by tail tip bleed. Six days after i.p. inoculation with blood-stage *P. berghei* ANKA, C57BL/6 mice exhibited peripheral blood parasitemia of 7.41% (IQR: 5.23-9.68).

Blood and Tissue Sample Collection

On day 6 post-inoculation, mice were terminally anesthetized and 700 µl of blood was collected. To prevent coagulation, the syringe contained 60 µl DPBS with K_3EDTA (final concentration of 1.6mg/ml K_3EDTA after mixing with 700 µl of blood). 100 µl of whole blood was removed for nitrite analysis. Plasma was separated from erythrocytes by immediate centrifugation at 3,000×G for 5 minutes at 4°C, snap-frozen on dry ice, and stored at -80°C until analysis. Remaining blood was removed from the vascular compartment by transcardial perfusion with 20 ml 1×PBS. Lung, liver, kidney, spleen, aorta, heart and whole brain were dissected, removed, snap-frozen on dry ice and stored at -80ºC until analysis.

Quantitative RT-PCR

RNA was extracted from Trizol preserved liver tissue using Qiagen RNeasy kit (Qiagen, Valencia, CA, USA). RNA was reverse transcribed in the presence or absence of reverse transcriptase using Invitrogen Superscript® VILO™ MasterMix (Life Technologies, Carlsbad, CA, USA), followed by quantitative PCR using SensiMix SYBR® Green PCR Mix (Bioline, Taunton, MA, USA). Primer pairs 5'-CATGTCTTGCTGCACCGAAC-3' and 5'-GACCTTTGCGCTTTCTGG-3' were used to amplify DDAH1. Mouse GAPDH was simultaneously amplified as an internal control, using primer sequences 5′-CATCACCATCTTCCAGGAGCG-3′ and 5′-GAGGGGCCATCCACAGTCTTC-3′ [64].

Western Blot

Liver samples were thawed and homogenized in 1X PBS containing protease inhibitor cocktail (Roche Applied Sciences, Indianapolis, IN) using a Lab Gen 7 series hand held homogenizer (Cole Parmer, Vernon Hills, IL, USA). Homogenates were centrifuged for 5 min at 16,100 ×G and 4°C and supernatants were removed and placed on ice. Protein concentrations were determined with a Pierce BCA Protein Assay Kit (Thermo Scientific, Sunnyvale, CA, USA) according to the manufacturer's instructions. Protein homogenates (50 µg) were mixed 1:1 with 2X SDS sample buffer (Life Technologies, Carlsbad, CA, USA) and heated at 95° C for 10 min. Proteins were separated on a 4-12 % SDS-polyacrylamide gel (Life Technologies, Carlsbad, CA, USA) at a constant voltage (90-100 V) and transferred to nitrocellulose membrane with an iBlot transfer apparatus (20V for 8min) (Life Technologies, Carlsbad, CA, USA). The membrane was incubated for 1h in a blocking buffer consisting of 5% nonfat dry milk and 0.1 % Tween 20 in 1×PBS (Lonza, Walkersville, MD, USA). The membrane was then incubated overnight in blocking buffer containing 1:3000 anti- DDAH1 (Sigma-Aldrich, St. Louis, MO, USA) at 4° C with constant rocking. After washing with PBS containing 0.1 % Tween-20, the membrane was incubated for 1h in blocking buffer containing 1:10,000 anti-rabbit-HRP secondary antibody (GE Healthcare UK Ltd.). The membrane was then washed with 1×PBS, treated with West Femto substrate (Pierce) and visualized with CL-Xposure blue X-ray film (Thermo Scientific, Sunnyvale, CA, USA). Membranes were stripped and re-probed with an anti-ß actin-HRP (Abcam, Cambridge, MA, USA) antibody as a control.

DDAH Enzyme Activity Assay

Frozen liver samples were homogenized in 8 µl 1×PBS (Lonza, Walkersville, MD, USA) per mg wet weight of tissue with a Lab Gen 7 series hand held homogenizer (Cole Parmer, Vernon Hills, IL, USA). Homogenized samples were centrifuged for 5 min at 16,100×G and 4°C to remove insoluble material. Supernatants were diluted 1:5 in 1×PBS spiked with ADMA (Cayman Chemical, Ann Arbor, MI, USA) for a final concentration of 2.5 mM ADMA. Spiked homogenates were mixed and sampled immediately (0 hr time point) and after 2 hr at 37°C. At each time point, citrulline was quantified by mixing 15 µl of homogenate with 15µl of 2M perchloric acid containing 25 µmol/L N^G-monoethyl-L-arginine (MEA) as an internal standard. Samples were centrifuged for 5min at 16,100×G and 4°C to remove precipitate, and 10 µl of the supernatant was neutralized with 5µl 1.5M potassium carbonate (Sigma-Aldrich, St. Louis, MO, USA) in a fresh tube. Neutralized samples were further diluted 1:2 in ultrapure water and centrifuged to remove potassium perchlorate precipitate. HPLC was performed on a low-flow Agilent 1100 series instrument and Agilent Chemstation OpenLab CDS revision C.01.03 was used to control the instrument and acquire data. Samples were derivatized directly prior to analysis by programming the autosampler to draw 2µl of derivatization reagent, 2µl of sample, mix for 30s and inject. The derivatization reagent contained final concentrations of 1.5mg/ml (11.2 mM) ortho-phthalaldehyde (Sigma-Aldrich, St. Louis, MO, USA) and 41.3mM 3-mercaptopropionic acid (Sigma-Aldrich, St. Louis, MO, USA) diluted in 200mM potassium tetraborate solution (pH 9.4). Citrulline and the MEA internal standard were separated with a 1×100 mm C18(2) column with 3 µm particles and 100Å pore size (Phenomenex, Torrance, CA, USA). Solvent A was composed of 0.1M sodium acetate (Fisher Scientific, Pittsburgh, PA, USA), 9 % v/v methanol (Fisher Scientific, Pittsburgh, PA, USA) and 0.5 % v/v tetrahydrofuran (Sigma-Aldrich, St. Louis, MO, USA) in ultrapure water (Milli-Q Synthesis system, Millipore Corporation, Billerica, MA, USA) with pH adjusted to 8.3 with 3M NaOH (Sigma-Aldrich, St. Louis, MO, USA). Organic mobile phase (Solvent B) was HPLC-grade methanol (Fisher Scientific, Pittsburgh, PA, USA) and separation of analytes was accomplished with a gradient from 0% solvent B to 25 % solvent B over 20min at a flow-rate of 70 µl/min. The column was then washed with 100% solvent B for 5 min and re-equilibrated with 100% Solvent A for 20 minutes. The column was maintained at 40°C for the duration of the run. Citrulline and the MEA internal standard were quantified by fluorescence detection using excitation and emission wavelengths of 340 nm and 455 nm respectively. Citrulline production was calculated by taking the difference of starting (0 hr) and ending (2 hr) concentrations, dividing by the 2 hr time interval and normalizing to protein content as quantified by BCA colourimetric assay (Thermo Scientific, Sunnyvale, CA, USA) performed according to the manufacturer's instructions (Figure S5).

Determination of Tissue ADMA

Frozen liver samples were homogenized in 8 µl PBS per mg wet weight of tissue with a Lab Gen 7 series hand held homogenizer (Cole Parmer). Homogenized samples were centrifuged for 5 min at 16,100 × G and 4°C to remove insoluble material. An aliquot of supernatant was stored for protein quantification and a second was precipitated by mixing 1:1 with 2 M perchloric acid (Fisher Scientific) containing monoethylarginine (Enzo Life Sciences) as an internal standard. Precipitated samples were centrifuged for 5 min at 16,100×G and 4°C and the resulting supernatant was removed and neutralized with 4 volumes of 0.5M Na_2HPO_4. Neutralized samples were extracted with Oasis MCX 96-well µElution Plates (Waters Corporation) and analyzed with HPLC conditions as described above for determination of plasma concentrations of arginine and ADMA with the following exceptions: 1) chromatography was performed on a 1×100 mm C18(2) column with 3µm particles (Phenomenex) at a flow-rate of 90 µl/min; 2) solvent A consisted of 25 mM sodium phosphate and 5% acetonitrile; 3) separations were accomplished by 20 min of isocratic conditions followed by a gradient from 0%B at 20 min to 18%B at 42 min. Protein was quantified in liver homogenate supernatants with the BCA colorimetric assay (Pierce) and used to normalize ADMA concentrations to total liver homogenate protein.

Determination of Arginine and ADMA in Mouse Plasma

Mouse plasma samples were extracted as described above and analyzed using an HPLC assay modified from the method described above for analysis of human samples. Analysis of mouse plasma

samples were performed on a low-flow Agilent 1100 series HPLC instrument and Agilent Chemstation OpenLab CDS revision C.01.03 was used to control the instrument and acquire data. The autosampler was programmed to draw 1.7µl of ortho-phthalaldehyde (OPA) derivatization solution and 1.7µl of the current sample in the sequence, mix the contents in the needle for 30s and inject the derivatized sample onto the column. OPA derivatization reagent contained 7.46 mM ortho-phthalaldehyde (Sigma-Aldrich, St. Louis, MO, USA) and 27.5 mM 3-mercaptopropionic acid (Sigma-Aldrich, St. Louis, MO, USA) in 200 mM potassium tetraborate (Sigma-Aldrich, St. Louis, MO, USA), pH 9.4. Chromatography was performed on a 1×100 mm C18(2) column with 3 µm particles and 100 Å pore size (Phenomenex, Torrance, CA, USA) at a flow-rate of 90 µl/min. Solvent A consisted of 25 mM sodium phosphate and 5% acetonitrile in ultrapure water and solvent B consisted of 50% acetonitrile in ultrapure water. Separations were accomplished by 20 min of isocratic conditions followed by a gradient from 0%B at 20 min to 18%B at 42 min. The column was then washed with 100 % solvent B for 5 minutes and re-equilibrated for 13 minutes prior to injection of the following sample. The column was maintained at 35°C for all runs. Online fluorescence was measured at excitation and emission wavelengths of 340nm and 455nm, respectively. The fluorescence detector's photomultiplier tube (PMT) gain was set to 11 for initial normal-sensitivity analysis. At 20min, PMT gain was switched to 16 for the detection of ADMA. At 37 minutes PMT gain was switched back to 11 for detection of the MEA internal standard with normal sensitivity. Chemstation OpenLab CDS software version C.01.03 was used for peak integration. Analyte peaks were quantified by comparison to the internal standard (MEA).

Whole Blood Nitrite Immediately after collection, 100µl of whole blood was mixed with 25µl of a solution containing potassium hexacyanoferrate(III) (800mM) (Sigma-Aldrich, St. Louis, MO, USA) to oxidize ferrous heme, N-ethylmaleimide (100mM) (Sigma-Aldrich, St. Louis, MO, USA) to block thiols and 10% v/v Nonidet-40 substitute (Fisher Scientific, Pittsburgh, PA, USA) to solubilize cell membranes. Samples were promptly snap-frozen on dry ice and stored at -80°C until analysis. For analysis, whole blood samples were thawed and deproteinated by mixing 1:1 with methanol (EMD Millipore, Billerica, MA, USA) and centrifuging for 5min at 16,000×G and 4°C. Supernatants were injected into a reaction chamber containing potassium iodide (66.9mM) and iodine (28.5mM) (Sigma-Aldrich, St. Louis, MO, USA) diluted in a 2:7 mixture of ultrapure water (Milli-Q Synthesis System, Millipore Corporation, Billerica, MA, USA) and acetic acid, glacial (Fisher Scientific, Pittsburgh, PA, USA). NO produced by reduction of nitrite in plasma samples was detected with a Nitric Oxide Analyzer 280i (GE Healthcare, Pittsburgh, PA, USA) and compared with standards of known concentration prepared from sodium nitrite (Sigma-Aldrich, St. Louis, MO, USA). Data processing and peak integrations were performed with OriginPro 8 software.
